# Uncovering Genomic Regions Associated with *Trypanosoma* Infections in Wild Populations of the Tsetse Fly *Glossina fuscipes*

**DOI:** 10.1534/g3.117.300493

**Published:** 2018-01-17

**Authors:** Andrea Gloria-Soria, W. Augustine Dunn, Xiaoqing Yu, Aurélien Vigneron, Kuang-Yao Lee, Mo Li, Brian L. Weiss, Hongyu Zhao, Serap Aksoy, Adalgisa Caccone

**Affiliations:** * Department of Ecology and Evolutionary Biology, Yale University, New Haven, Connecticut 06511; † Department of Biostatistics, Yale School of Public Health, New Haven, Connecticut 06511; ‡ Department of Epidemiology of Microbial Diseases, Yale School of Public Health, New Haven, Connecticut 06511

**Keywords:** gene–phenotype association in tsetse flies, *Trypanosoma*, sleeping sickness, population genomics

## Abstract

Vector-borne diseases are responsible for > 1 million deaths every year but genomic resources for most species responsible for their transmission are limited. This is true for neglected diseases such as sleeping sickness (Human African Trypanosomiasis), a disease caused by *Trypanosoma* parasites vectored by several species of tseste flies within the genus *Glossina*. We describe an integrative approach that identifies statistical associations between trypanosome infection status of *Glossina fuscipes fuscipes* (*Gff*) flies from Uganda, for which functional studies are complicated because the species cannot be easily maintained in laboratory colonies, and ∼73,000 polymorphic sites distributed across the genome. Then, we identify candidate genes involved in *Gff* trypanosome susceptibility by taking advantage of genomic resources from a closely related species, *G. morsitans morsitans* (*Gmm*). We compiled a comprehensive transcript library from 72 published and unpublished RNAseq experiments of trypanosome-infected and uninfected *Gmm* flies, and improved the current *Gmm* transcriptome assembly. This new assembly was then used to enhance the functional annotations on the *Gff* genome. As a consequence, we identified 56 candidate genes in the vicinity of the 18 regions associated with *Trypanosoma* infection status in *Gff*. Twenty-nine of these genes were differentially expressed (DE) among parasite-infected and uninfected *Gmm*, suggesting that their orthologs in *Gff* may correlate with disease transmission. These genes were involved in DNA regulation, neurophysiological functions, and immune responses. We highlight the power of integrating population and functional genomics from related species to enhance our understanding of the genetic basis of physiological traits, particularly in nonmodel organisms.

The tsetse fly, *Gff*, is an important vector of both human (HAT, sleeping sickness) and animal (AAT, Nagana) Trypanosomiasis in Africa. Trypanosome transmission is complex, as it requires mammalian and invertebrate hosts, and involves domestic and wild reservoirs. *Gff* is the main HAT vector in Uganda, the only country where the two types of HAT-causing parasites cooccur. More specifically, *Trypanosoma brucei gambiense*, which causes the chronic form of HAT, is found in northwestern Uganda, while *T. brucei rhodesiense*, which causes the acute form of the disease, occurs in the country’s southeast. Although the range of the two diseases is geographically separated, the gap between them is rapidly narrowing and likely to merge in the near future in northern Uganda (Picozzi *et al.* 2005). Because the pathology, diagnosis, and treatment varies between the two disease forms, an eventual overlap of the two disease belts would complicate HAT control (Welburn *et al.* 2009).

Previous work from our laboratory, using microsatellite loci and mitochondrial DNA, has documented spatial and temporal patterns of genetic variation, and levels of genetic differentiation within and among populations of *Gff* in Uganda. Three genetically distinct *Gff* population clusters can be identified: the northern, western, and southern regions (Beadell *et al.* 2010; Hyseni *et al.* 2012). These clusters are both spatially and temporally stable (Beadell *et al.* 2010; Echodu *et al.* 2011; Hyseni *et al.* 2012; Opiro *et al.* 2016, 2017), even though hybrid zones among these units exist (Beadell *et al.* 2010; Opiro *et al.* 2017), which facilitate genetic admixing within a 100 km radius (Beadell *et al.* 2010). This information can be used to guide vector monitoring and control interventions. However, it does not provide insights on the genetic basis of traits shaping the interactions between vectors and parasites.

Understanding the interaction between vectors and their parasites is fundamental to inform disease control methods, but data from natural populations is scarce. This is a major knowledge gap with important epidemiological implications. To facilitate the study of genotype–phenotype interactions, we recently expanded the genetic marker toolset of *Gff* using whole-genome sequencing (WGS) coupled with double digestion RAD methods [ddRADSeq (Baird *et al.* 2008; Peterson *et al.* 2012)] to a set of ∼73,000 SNPs distributed across the *Gff* genome (Gloria-Soria *et al.* 2016). The *Gff* SNP data set obtained from this pilot study was generated from flies belonging to each of the three Ugandan genetic clusters (*N* = 53), with the same number of infected and uninfected flies per population. This data set was used to calculate the average linkage disequilibrium (LD) across the *Gff* genome and to look for signatures of selection via genomic scans (Gloria-Soria *et al.* 2016). Our results indicated that LD in *Gff* decays at a slower rate than in other insects, being an order of magnitude lower than in the fruit fly *Drosophila melanogaster* (Mackay *et al.* 2012). The average genomic LD also varied among the *Gff* populations (*r*^2^_max_/2 ranged between 1359 and 2429 bp) (Gloria-Soria *et al.* 2016). These high levels of LD likely increased our ability to subsequently identify loci under selection, despite the small sample size (Gloria-Soria *et al.* 2016). The results of this pilot study support the feasibility of conducting GWAS analyses in this species, using much lower sample sizes that those required by other species, including *Anopheles* or *Aedes* mosquitoes, whose LD levels are quite low (Harris *et al.* 2010; David *et al.* 2014; Marsden *et al.* 2014).

Although the pilot study yielded a list of candidate SNPs, mostly related to environmental adaptation (Gloria-Soria *et al.* 2016), only one SNP was identified as associated with the infection status of the flies using outlier detection methods (Fischer *et al.* 2011; Duforet-Frebourg *et al.* 2014). Furthermore, functional characterization of candidate genes was complicated by the poor annotation of the *Gff* genome and the lack of transcriptomics for this species, a common problem in nonmodel organisms. The present work extends the analyses on the initial ddRAD data set from Gloria-Soria *et al.* (2016) to gain additional insights on the nature of the genomic regions associated with the susceptibility to *Trypanosoma* infection of *Gff* populations by using statistical methods that maximize efficiency and power (Berg and Coop 2014), while accounting for spatial stratification of the samples as well as for species- and population-specific LD (Purcell *et al.* 2007). This work takes advantage of the functional data available from a closely related species, *Gmm* (Petersen *et al.* 2007), to improve the *Gff* genome annotation of candidate genes, thus enabling their functional characterization. Comparison of the genomes from six tsetse fly species, including *Gmm* and *Gff*, is ongoing, but our preliminary analysis indicates that > 90% of the predicted coding sequences from *Gff* align to the *Gmm* sequences. The remarkable similarity across the *Gff* and *Gmm* genomes highlights the validity of the present approach. As a result of this method, we identified candidate parasite resistance genes in *Gff* that could potentially be used to develop targeted control strategies for this and other *Glossina* species, paving the way for association studies on other phenotypes of epidemiological interest in this and other tsetse species (*e.g.*, developmental time, life span, and susceptibility to insecticides, *etc*.).

## Materials and Methods

### Gff association analyses

#### Gff genomic data:

The starting SNP data set for this study included all 73,297 loci identified by Gloria-Soria *et al.* (2016), using a combination of double digest RADseq (Peterson *et al.* 2012) and WGS (40× coverage/individual). The raw data are available from NCBI bioproject 303153. The SNPs are distributed across the *Gff* genome at a density of ∼1 SNP for every 5 kb and were derived from four geographically distinct populations in Uganda—Otuboi, OT (North), Masindi, MS (West), and Namutumba, NB and Kalangala Island (South)—which belong to three genetic clusters. Sampling details can be found in Gloria-Soria *et al.* (2016). The use of genetically distinct populations maximized the natural genetic diversity represented in the data set, while also minimizing ascertainment bias, thus reducing the impact of false positives in our analysis (Schoville *et al.* 2012).

#### Genetic association analysis for susceptibility to trypanosome infection:

The analysis was conducted on individuals from three of the four *Gff* populations from Uganda described in Gloria-Soria *et al.* (2016). These populations represent each of the three *Gff* genetic clusters in this country. The Kalangala Island population was excluded due to small sample size. Association analysis was performed using PLINK v1.9 (Chang *et al.* 2015) under a logistic regression model to identify SNPs associated with infection status. Infection status is treated as response and the predictor is the additive allele dosage effect of each marker. To minimize the confounding effects due to population structure, we used Faststructure v1.0 (Raj *et al.* 2014) to characterize the population structure by a vector (X1, X2, and X3), where the optimal dimensionality was determined by chooseK.py. Because (X1, X2, and X3) is compositional, log transformations were applied on X1/X3 and X2/X3 before including them in the model. We used this method because it can automatically select the complexity of the population structure (*i.e.*, the dimensionality of covariates), and provides more efficient computation and better interpretations of global ancestry (Raj *et al.* 2014). In addition, log transformation is a common statistical practice to deal with composition variables (Aitchison 1982) and has been used to identify covariates in association studies in order to account for population structure (Mariette *et al.* 2016). We also carried out a similar association analysis accounting for population structure via principle components (PCs). Specifically, the top two PCs were extracted (–pca in PLINK v1.9) and included in the logistic regression model.

Statistical evidence of association between disease status and each marker was assessed through the Benjamini–Hochberg-adjusted p-values (Benjamini and Hochberg 1995). Subsequently, since the distribution of association p-values did not fit the expected uniform distribution in the tailed portion of the histogram (Supplemental Material, Figure S1A), we implemented an alternative strategy to simplify the underlying complexity of the statistical analysis; that is, we extracted representative SNPs based on different LD thresholds (*r*^2^ = 0.01–0.001). This strategy aims to facilitate the interpretation of associations across regions.

### Harnessing Gmm genomic data

#### Gmm genomic data:

To functionally characterize the gene regions in *Gff* likely to be in association with *Trypanosoma* infection, we took advantage of the genomic and functional studies in a closely related species, *Gmm*, which is amenable to laboratory work (Petersen *et al.* 2007). We combined old and new transcriptome (step 1) and RNAseq (step 2) data to improve the published *Gmm* transcriptome assembly (step 3), then we generated a list of expressed *Gmm* transcripts expressed in various *Gmm* tissues (step 4), and ultimately identified those DE genes in relation to *Gmm Trypanosoma* infection (step 5); see Figure S2.

##### Step 1: Gmm transcriptome data:

Methods from previously published transcriptomes can be found in the corresponding references listed in Table S1. Unpublished transcriptomes followed the same overall protocol. Briefly, *Gmm* were maintained at the Yale School of Public Health insectary and infected with *T. brucei rhodensiense* (Ytat 1.1), as described previously (Aksoy *et al.* 2016). Fourteen days postchallenge, tissues were microscopically scored for the presence or absence of trypanosome infections, and tissues of interest were flash-frozen in liquid nitrogen for RNA extraction (see Table S1 for tissues processed). Total RNA was extracted using TRIzol (Invitrogen) and subjected to DNase treatment (TURBO DNA-free kit AM1907; Ambion). RNA quantity and quality were determined using an Agilent Bioanalyzer 2100 RNA Nano chip. cDNA libraries were prepared using a NEBNext Ultra Directional RNA Library Prep Kit (E7420S; New England Biolabs), according to the manufacturer’s protocol. The libraries were constructed and barcoded for Illumina HiSeq sequencing (unpaired 75 bases) at the Yale Center for Genome Analysis (New Haven, CT).

##### Step 2: Gmm RNAseq data:

The RNAseq data analysis pipeline is shown in Figure S2. The raw Illumina RNAseq reads from 72 samples (Table S1) were first assessed for quality by FastQC (Andrews 2010). The FASTX-toolkit (*http://hannonlab.cshl.edu/fastx_toolkit/*) was then used to trim off Illumina adapter sequences and low-quality bases. Due to the prevalence of tsetse symbiont sequences in the reads, a specific quality control step was included to reduce bacterial sequence reads, using reference genome data sets from the three symbionts that are most commonly found in tsetse flies (Aksoy *et al.* 2014): *Wigglesworthia* (Rio *et al.* 2012), *Wolbachia* (Brelsfoard *et al.* 2014), and *Sodalis* (Toh *et al.* 2006). Parasite sequences were removed from parasitized samples by mapping the Illumina reads to the *T. brucei* genome strain 927 and *T. congolense* (*www.tritrypdb.org*) prior to analysis. Reads passing quality control, and symbionts and parasites separation, were aligned to a previously generated contig library (Telleria *et al.* 2014) by TopHat2 (Kim *et al.* 2013) and Bowtie (Langmead *et al.* 2009) alignment engines. Reads that mapped to multiple locations were discarded from further analysis. The percentage of reads aligned to bacteria, parasites, and the host genome is shown in Figure S3. The raw Illumina RNAseq reads can be found under the accession numbers listed in Table S1.

##### Step 3: updating Gmm transcriptome assembly:

We constructed a more comprehensive *Gmm* transcriptome assembly than the one available through VectorBase [VectorBase (Giraldo-Calderón *et al.* 2015); GmorY1.4] by integrating the previous assembly with the 72 RNAseq samples described in Table S1. The reference annotation from VectorBase (Giraldo-Calderón *et al.* 2015) (GmorY1.4) was used to guide transcript assembly by Cufflinks v2.2.1 (Roberts *et al.* 2011a; Trapnell *et al.* 2012) and obtain the relative expression of a transcript (FPKM; fragments per kilobase of exon per million fragments mapped) for each annotated gene. The resulting 72 individual Cufflinks assemblies were then merged into a single unified assembly (*Gmm Tx*) using Cuffmerge, which can maximize assembly quality by removing transcripts that are artifacts and merging novel isoforms with known isoforms across all Cufflinks assemblies. This unified assembly was then integrated with the previous assembly (GmorY1.4), using Cufflinks to compare and determine a unique set of isoforms for each transcript locus.

##### Step 4: Gmm DE analysis:

Two strategies, a count-based method and Cufflinks pipeline, were used to identify DE genes between *Trypanosoma*-infected and noninfected *Gmm* samples. In the count-based strategy, raw gene counts were first counted by HTSeq package (Anders *et al.* 2015) using the unified assembly generated as above. Batch effects and other unwanted variations were removed using the RUVseq package (Risso *et al.* 2014). Batch effect-corrected counts were then normalized using the TMM method (Robinson and Oshlack 2010) in the edgeR package (Robinson and Smyth 2007, 2008; Robinson *et al.* 2010, 2012; Anders *et al.* 2013), which accounts for RNA composition bias.

##### Step 5: DE genes between Gmm Trypanosoma-infected and noninfected flies:

A negative binomial generalized linear model implemented in edgeR was used to determine DE genes between *Trypanosoma*-infected and noninfected samples. In the Cufflinks strategy, the abundance of transcripts was quantified by FPKM and compared between *Trypanosoma*-infected and noninfected samples using Cuffdiff with fragment bias correction (Roberts *et al.* 2011b). This method corrects for sequence-specific bias and conducts a multihit read correction by dividing the value of a multimapped read between each map location based on a probabilistic model. In both strategies, genes with a fold-change > 2 and an FDR-controlled p-value < 0.05 were considered DE. For comparisons without biological replicates, only fold-change was used as a criterion.

### Combining genomic data from Gff and Gmm

To gain insights into the functional role of *Gff* gene regions associated with *Trypanosoma* infection, we used the *Gmm* database, which included DE genes between infected and noninfected flies to narrow down the list of genes identified by the association analyses in *Gff* (see above) to only those genes displaying differential expression between infected and noninfected *Gmm*. First, we mapped the new *Gmm* transcriptome assembly to the *Gff* genome, producing a hybrid *Gff* annotation (step A). We then identified regions near the representative SNP of a region found to be associated with *Trypanosoma* infection in *Gff*, and DE in infected and noninfected *Gmm* flies (step B):

#### Step A: cross-species transcript mapping:

We used Blat (v35) (Kent 2002) to map the newly generated *Gmm* transcriptome assembly (*Gmm Tx*) to the *Gff* genome (GfusI1), with options set to exclude overoccurring 11mers from initial match seeds (–ooc 11). Blat hits (the *Gmm* transcripts that matched to some sequences of the *Gff* genome) were filtered and retained for further analysis if the hit was ≥ 100 bp long and had match coverage of ≥ 90%. Note that the purpose of using *Gmm Tx* was to supplement the current *Gff* annotation with genomic regions that may be missing, not to “fix” existing *Gff* annotations. For this reason, only *Gmm Tx* that did not overlap existing annotated features in *Gff* were added to the original *Gff* annotations to generate the final set of annotations used in our downstream analysis.

#### Step B: determination of DE genes in Gff linked to the SNPs in association with trypanosome infection:

After identifying *Gff* regions that shared > 90% sequence identity with annotated *Gmm* genes and were longer than ∼120 bp, we added any novel *Gff*-annotated region to a hybrid gene annotation for *Gff*. We then looked in this hybrid annotation for genome features within 2500 bp in either direction of each representative SNP associated to trypanosome infection status. This distance was determined based on previous LD findings (Gloria-Soria *et al.* 2016). We then cross-checked these gene regions in *Gff* with the *Gmm* data (above) to identify those DE in *Gmm*. The infection-associated DE genes in *Gmm* were used to filter/annotate the *Gff* hybrid annotation for probable trypanosome infection-related *Gmm* homologs. This step helped to focus our efforts on candidates with a confirmed biological function (“real” genes as opposed to possible annotation artifacts) based on their DE expression in *Gmm*.

### Functional characterization of candidate Gff genes

*Drosophila* and tsetse flies are both higher Dipterans that share several biological pathways. Previous studies investigating tsetse gene functions at the molecular level have shown a high conservation between tsetse flies and *Drosophila*, especially with regard to their interaction with microbes (Attardo *et al.* 2014; Benoit *et al.* 2017). Thus, to gain insight into the functions of our candidate genes, we looked for their homologs in these species. Transcripts from the *Gff* genes associated with trypanosome infection status were ascertained using the annotated VectorBase (*www.vectorbase.org*) *Gff* transcript database Gfus1.4. These resulting transcripts were then compared to the VectorBase *Gmm* transcript database GmorY1.5 using the VectorBase BLASTx tool to determine their corresponding homologous transcripts in *Gmm*. Similarly, the same subset of *Gff* transcripts were blasted (BLASTx) to FlyBase (*www.flybase.org*) to identify their corresponding *D. melanogaster* homologs.

### Data availability

**Figure 1:**
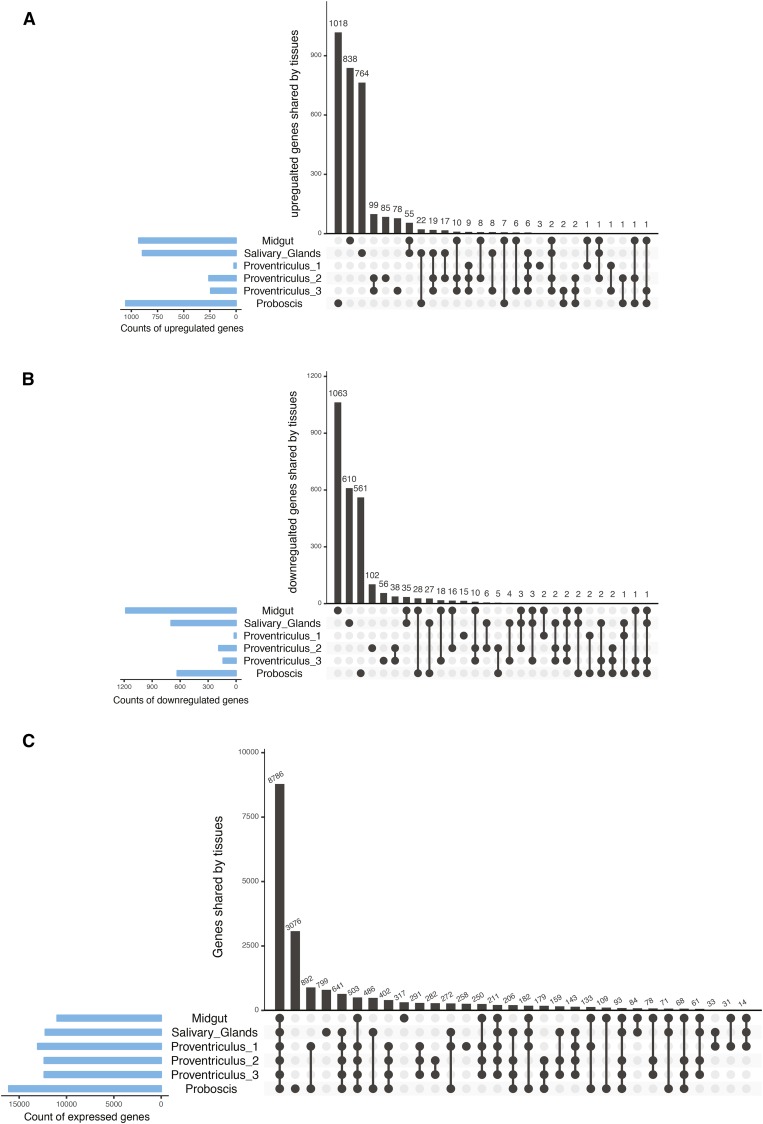
Gene expression across *G. morsitans morsitans* (*Gmm*) tissues. (A) Upregulated and (B) downregulated genes in infected *Gmm* tissues. (C) Genes expressed across tissues. The count of genes identified in any more than one tissue is plotted [differentially expressed genes (DE), as identified by both cuffdiff and edgeR]. Dark circles in the bottom matrix indicate tissues that share the same set of genes. The number of genes in each tissue are shown as blue bars at the bottom left. A total of 19,110 unique genes were expressed in at least one tissue, with 8786 (46%) expressed across all six tissues.

Original ddRAD data are available from NCBI bioproject 303153. Table 1 provides a list of the *Gff* genes associated with trypanosome infection that have homologs differentially expressed among *Gmm*-infected and noninfected flies. Table S1 provides information on the 72 *Gmm* transcriptomes used in this study and their corresponding SRA accession numbers. Table S2 and Table S3 provide the SNPs associated with trypanosome infection status in *Gff* using two alternative methods to control for population structure. Table S4 provides a comparison between the new and previous *Gmm* transcriptome assembly. Table S5 contains the genes DE between infected and uninfected *Gmm* tissues. Table S6 describes the multiple genome feature IDs used throughout the paper and association across the ID types. Table S7 lists the 56 candidate genes identified in the vicinity of representative SNPs associated with trypanosome infection status. Table S8 compares the DE genes in *Gmm* across tissues. Table S9 provides the results from comparing the candidates SNPs from Gloria-Soria *et al.* (2016) with those found in this study. Figure 1 shows the DE genes shared across tissues for *Gmm* and the number of genes expressed by tissue. Figure S1 shows the Manhattan plot before and after clumping the association results based on LD. Figure S2 shows the analysis pipeline of the *Gmm* transcriptome. Figure S3 provides the percentage of transcriptome reads from *Gmm* aligned to the host, bacteria, and parasite genomes. Figure S4 shows the Manhattan plots and histograms of p-values from association for each population of *G. fuscipes* clumped at an LD threshold of 1%.

**Table 1:** *Gff* genes associated with trypanosome infection that have differentially expressed homologs among *Gmm* infected and noninfected flies

Functional Category	Gene Identifier*^a^*	*G. morsitans morsitans* Homolog*^b^*	BLAST Result Against *Drosophila* Database*^c^*	*Drosophila* Annotation Symbol*^d^*	Potential Function*^e^*	References*^f^*
Neurophysiology	GFUI053401	GMOY003473	Protein Phosphatase 99A (Ptp99A)	CG11516	Neural receptor, potential target of miR-8, in *Drosophila* presents a SNP associated with microbiota-dependent nutriments trait	Desai *et al.* (1997), Enright *et al.* (2003), Dobson *et al.* (2015)
	GFUI021833	GMOY011124	Protein tincar	CG31247	Expressed during *Drosophila* embryogenesis in central/peripheral nervous systems and midgut	Hirota *et al.* (2005)
	GFUI017734	GMOY004860	Malvolio	CG3671	Required for normal taste behavior in *Drosophila*, expressed in the central nervous system, macrophages, and gut of *Drosophila*, Expression of Malvolio correlates with feeding habits preferences in Calliphoridae	Rodrigues *et al.* (1995), Folwell *et al.* (2006), Cardoso *et al.* (2016)
	GFUI041241	GMOY006879	Insulin-like peptide	CG14059	Feeding regulation, growth and development in *Drosophila*	Pool and Scott (2014), Garelli *et al.* (2012)
	Novel	GMOY009338	*Drosophila* IGF-II mRNA-binding protein (dimp)	CG9850	Involved in nervous system development, promotes neuronal remodeling	Adolph *et al.* (2009), Medioni *et al.* (2014)
	GFUI019498	GMOY008359	Innexin 7 (inx7)	CG2977	Gap junctions involved in nervous system	Liu *et al.* (2016)
	Novel	GMOY005501	Synaptic vesicle glycoprotein 2B-like	CG31106	Homologous gene in cat flea, expression in parallel to feeding process, modulation of synaptic exocytosis in vertebrates	Walmsley and Gaines (2004), Morgans *et al.* (2009)
Cell proliferation and differentiation	GFUI009421	GMOY009422	Winged eye	CG31151	Regulation of *histone* gene expression	Ozawa *et al.* (2016)
	GFUI043027	GMOY005878	DNA polymerase ɛ subunit 2 (pole2)	CG10489	Involved during the progression of mitosis (S phase)	Sahashi *et al.* (2013)
	GFUI023751	GMOY005801	Cytoskeleton-associated protein 5 (msps-like)	CG5000	Essential for structural integrity of mitotic spindle	Cullen *et al.* (1999)
	GFUI043720	GMOY007600	Cell division cycle 14	CG7134	Controls late cycle of mitosis	Stegmeier and Amon (2004)
	GFUI002252	GMOY004487	Moira	CG18740	Chromatin-remodeling factor, associated with cell cycle and cell proliferation	Crosby *et al.* (1999), Nakamura *et al.* (2008)
	GFUI030827	GMOY002286	Wee1-like kinase	CG4488	Regulator of *Drosophila* embryo mitosis	Stumpff *et al.* (2005)
	GFUI011175	GMOY009522	Adenomatous polyposis coli (APC)	CG1451	Intestinal stem cell proliferation, regulation of Wnt pathway	Lee *et al.* (2009), Kunttas-Tatli *et al.* (2015)
Immune response	GFUI007441	GMOY004477	Dorsal	CG6667	Main transcription factor of the insect immune response Toll signaling pathway	Valanne *et al.* (2011)
	Novel	GMOY007569	Toll-like	CG7250	Main receptor of the innate immunity Toll signaling pathway	Lemaitre *et al.* (1996)
	GFUI020794	GMOY007570	Peroxiredoxin 6005	CG3083	Antioxidant, probably cytosolic in *Glossina morsitans*	Munks *et al.* (2005)
Miscellaneous	GFUI020790	GMOY007571	acyl-CoA-binding protein	CG8814		
	GFUI051070	GMOY001698	SET and MYND domain-containing, arthropod-specific, member 4 (Smyd4)	CG11160	Muscle development in *Drosophila*	Thompson and Travers (2008)
	GFUI043025	GMOY005879	Focadhesin	CG3520		
	GFUI028910	GMOY008384	Serine hydrolase	CG5707		
	GFUI020792	GMOY007568	Congested-like trachea (colt)	CG3057		
	Novel	GMOY005234	PFTAIRE-interacting factor 1A	CG42599		

a *G.fuscipes* gene ID in Vector Base. "Novel" refers to transcripts that have been characterized in this study.

b *G. morsitans* (*Gmm*) closest homolog gene ID in Vector Base

c The closest *Drosophila* homologs determined using BLASTx and displayed by their name and

d annotation symbol.

e Potential functions are listed along with the ^f^corresponding references.

## Results

### Associations between genetic variation and trypanosome infection status in Gff

We used the 73,297 SNPs previously developed for *Gff* (Gloria-Soria *et al.* 2016) to identify markers associated with trypanosome infection status (see *Materials and Methods*). Association results were grouped based on LD clumping to facilitate interpretation of the association signal. We explored several LD thresholds (*r*^2^ = 0.01–0.001) and selected the data set with a null distribution of p-values closest to a uniform distribution (Figure S1). An LD threshold of *r*^2^ < 0.01 was chosen and yielded a set of 63 SNP groups centered around a representative SNP or “index variant” (PLINK; Chang *et al.* 2015) shown in Table S2. The resulting Manhattan plot and the corresponding histogram of p-values are displayed in Figure S1C. As shown across Figure S1, accounting for LD facilitated detection of the association signal. From the 63 representative SNPs, 18 displayed statistically significant associations with trypanosome infection status, after correction for multiple testing (FDR-controlled p-value < 0.05; Table S2A) and accounting for population structure using FastStructure (Raj *et al.* 2014).

As LD levels can vary among populations, similar analyses were carried out separately for each of the three *Gff* populations—OT, NB, and MS—with the goal of identifying relevant population-specific markers. For each population, LD clumping yielded ∼60 representative SNPs (threshold of *r*^2^ > 0.01; Table S2, B–D) and we identified 19, 19, and 16 SNPs in OT, NB, and MS, respectively, which were significantly associated with the phenotype (Table S2, B–D). The corresponding Manhattan plots and histograms of p-values for each population are shown in Figure S4.

Table S3 shows the results of the association analysis using PCs as covariates, as an alternative approach to FastStructure (Raj *et al.* 2014) to control for population structure. Results were similar to those using FastStructure (above), but yielded 64 representative SNPs during the clumping procedure (previously 63). Additionally, one of the previously identified representative SNPs showed significant association values using the PC method (GFvariants_VB2014e_tvcf:KK351818.1:1387926, pval = 0.0046 after FDR).

### Enhancing the Gff transcriptome assembly

In our effort to identify *Gff* genes relevant to the trypanosome infection status, we took advantage of the already existing *Gmm* transcriptome assembly [GmorY1.4 (Giraldo-Calderón *et al.* 2015)] and improved it with data from 72 additional RNAseq samples (Table S1). The new assembly (*Gmm Tx*) covers 41,174 genes with 85,849 transcripts, among which 12,053 are completely matched with previous assemblies and 38,632 are potentially novel isoforms. A comparison between the existing assembly and the one generated in the current study can be found in Table S4.

### DE genes in Gmm

DE genes were identified between *Trypanosoma*-infected and uninfected *Gmm* samples in four tissues [midgut, salivary glands, cardia (proventriculus), and proboscis], using both count-based methods and the Cufflinks pipeline (Table S5). Both differential expression analysis strategies identified a substantial portion of genes (12–15% in the midgut, 40–50% in the salivary glands, 10–40% in the cardia, and 50% in the proboscis) that were only annotated in the new assembly (Table S5A). One-third of the DE genes were identified with both edgeR and Cuffdiff methods (Table S5B). Figure 1A-B show the count of genes identified as being DE across tissues.

### Cross-species transcript mapping

The newly assembled *Gmm* transcriptome was then used to enhance *Gff* existing genomic features. Mapping the new *Gmm* transcriptome assembly (*Gmm Tx*) to the *Gff* genome resulted in 20,448 *Gmm* transcripts that matched to sequences in the *Gff* genome. Among them, 15,954 were already annotated in *Gff*. Another 4494 were mapped to the *Gff* genome and represent novel annotations. The vast majority of the filtered *Gmm Tx* (99.11%) mapped to a unique location on the *Gff* genome. Of the novel annotations, the maximum number of *Gmm Tx* mappings after filtering was 21, while the mean number of mappings for all *Gmm Tx* was 1.017 (median = 1). Details about the genome features used through this cross-species mapping can be found in Table S6.

### Candidate Gff loci involved in susceptibility to trypanosome infection

We identified 56 *Trypanosoma* infection-associated *Gff* loci (Table S7) by screening for genes 2500 bp upstream and downstream of the representative SNPs displaying a significant association signal with the *Trypanosoma* infection phenotype (SNPs across all populations [*N* = 18] and SNPs for each individual population [*N* = 19, 19, and 16 for OT, NB, and MS, respectively] (see association section above). We also looked for genes within a 2500 bp window around the additional SNP identified across populations, using the PCA method to control for population structure, but found none.

The 56 identified loci can be divided into two categories: “official” and “novel” *Gff* loci. The first category includes 42 loci that fall within official *Gff* (GFusI1.3) gene annotations and bear official gene symbols, prefixed with “GFUI.” The second category represent 14 *Gff* loci where no official annotation exists but where a transcript was inferred using cufflinks-deduced *Gmm Tx* transcripts longer than 100 bp (mapped with 100% identity over ≥ 90% of its length). These genes, added by virtue of the projection of homologous transcripts of *Gmm* onto the *Gff* genome, are referred to by a cufflinks-assigned ID and a “TCONS” prefix (see Table S6 for genome feature description). All official and novel loci reported here have two common characteristics; a SNP with a significant genetic association located within ≤ 2500 bp of its coordinates, and an ortholog (for official loci) or corresponding TCONS Tx (for novel loci) in *Gmm* (Table S7). Thus, 42 official *Gff* genes and 14 novel *Gff* genes (a total of 56 *Trypanosoma*-associated *Gff* loci) were identified by this selection criterion and are listed in Table S7. One of these novel genes (XLOC_035548) was not annotated in either of the two genomes. Twenty-nine of all 56 genes identified were DE between *Trypanosoma*-infected and uninfected *Gmm* flies (Table 1).

To evaluate whether DE of 29 out of 56 loci in *Gmm* was significant (not random), we determined how common DE was across tissues in *Gmm*. We identified the total number of unique DE genes across the *Gmm* tissues tested and evaluated whether the disagreement in DE was due to a difference in the genes expressed across tissues. Figure 1, A and B show the DE genes shared by tissue, identified by methods cuffdiff, edgeR (Table S5A), and both (identified by both cuffdiff and edgeR, Table S5B), respectively. Between 2000 and 7000 unique DE genes were identified by these methods, with no DE genes shared by all six tissues and only one-tenth shared by at least two tissues (Figure 1, A and B and Table S8). This disagreement in DE could arise from tissues associated with infection differentially or simply because different genes are expressed in different tissues. To address this issue, we compared all expressed genes (genes with at least two reads aligned in at least half of the samples) across tissues. The number of genes expressed by tissue is shown in Figure 1C. A total of 19,110 unique genes were expressed in at least one tissue, with 8786 (46%) expressed across all six tissues. These two findings together indicate that a large difference exists across tissues in terms of the genes associated with infection status, and suggests that finding 29 out of 56 DE loci in *Gff* is not likely due to DE being common across tissues.

### Functional characterization of candidate Gff genes found in association with Trypanosoma infection status

We functionally characterized the 29 candidate genes linked to the phenotype of interest (*Trypanosoma*-infected *vs.* noninfected flies) to which a DE *Gmm Tx* had been mapped. We used this criterion to reduce the possibility of annotation artifacts among our candidates, thus ensuring that only real genes were included. Furthermore, this specific data set of genes would more likely underlie differences in vector competency across both species, given that they are closely related. We searched for *D. melanogaster* homologs of these 29 *Gff* genes by BLASTx analysis of the ascertained *Gff* transcript sequences against the fruit fly genome database [FlyBase (Gramates *et al.* 2017)]. These 29 genes were assigned to 23 functional homologs in *Drosophila*, with five of the novel *Gff* genes being paralogs of *Gff* official genes (Table 1), and no functional homolog found for the XLOC_035548 gene. The XLOC_035548 (TCONS_00071717) sequence maps to *Gmm* scaffold “scf7180000652014” from position 87,226 to 87,796; the nearest gene is ∼20 kb upstream: CSP2 (GMOY010026). Further searching the PFAM-A database of protein domain models (Finn *et al.* 2016) yielded no known functional domains within the sequence. Table 1 summarizes these results, showing that the products of seven of the *Gff* genes identified are associated with insect neurophysiology, including putative roles in feeding behavior; seven gene products are associated with DNA regulation, involving mitosis and cell proliferation processes; three gene products are involved in fly immune responses; and six gene products do not fall within any discrete category.

### Comparison with previously identified SNPs associated with trypanosome susceptibility

The significant SNPs identified in the MS, NB, and OT populations, and the SNPs identified from the combined population set, were compared to the 360 SNPs previously reported as potentially associated with infection status using F_st_ outlier methods implemented in BayeScan (Gloria-Soria *et al.* 2016). A total of 55 SNP pairs were identified between the two SNP lists, based on the scaffolds they were annotated to. These SNP–SNP pairs and the distance between each pair are reported in Table S9. SNP 709851, located in scaffold 27 and identified in the MS population, was the only SNP identified in both studies as a candidate SNP. The other SNP pairs were located > 34 kb away from each other.

## Discussion

Performing genomic association analysis of disease susceptibility in nonmodel organisms is challenging due to the inability to perform controlled laboratory experiments, small sample sizes, and the lack of well-annotated genomes, or the absence of a reference genome to guide the efforts. Since the annotated *Gff* genome (Gfus1.3) was incomplete, we took advantage of additional genomic resources available for *Gmm*, a tsetse species for which there is extensive laboratory data (Petersen *et al.* 2007). Using transcriptome data from *Gmm*, we complemented missing annotations in the *Gff* genome, which allowed us to go beyond SNP associations to functionally identify candidate genes. Furthermore, we narrowed down our gene candidate list by identifying homologs that were DE among infected or uninfected *Gmm* flies that had been experimentally challenged with *Trypanosoma* (Table 1 and Table S5).

### Regions associated with trypanosome infection in Glossina spp

Tsetse flies of the genus *Glossina* are major vectors of human and nonhuman animal diseases caused by infection with African trypanosomes. Laboratory studies have identified genetic factors for resistance to trypanosomes in *Gmm* (Hamidou Soumana *et al.* 2017; Geiger *et al.* 2015), but no information is available on HAT vector species such as *Gff* (*e.g.*, host–parasite strain combinations) and in general for any species from natural populations. *Gff* colonies are difficult to maintain in the laboratory, making experimental manipulations in this species extremely challenging. Here, we tested the hypothesis that natural variation for resistance or sensitivity to *Trypanosoma* infection in wild populations of tsetse flies has a genetic basis and could be used to identify the genetic components underlying this epidemiologically important trait.

Preliminary analysis of the *Gff* SNP data set used for this study failed to identify candidate genes involved in susceptibility to trypanosome infection, based on allele frequency differences between treatment groups (Gloria-Soria *et al.* 2016). Such F_st_ outlier methods are powerful at detecting markers subjected to strong selection pressures, but have low power to detect markers under weak or balancing selection (Narum and Hess 2011; Jensen *et al.* 2016). Furthermore, the F_st_ outlier approach used in the pilot study, as implemented by BayeScan (Fischer *et al.* 2011), assumes that the contrasting populations are evolutionary independent from each other and that gene frequencies under neutrality approximate a multinomial Dirichlet distribution (Beaumont 2005). This method is generally appropriate to identify loci involved in environmental adaptation, and Gloria-Soria *et al.* (2016) successfully identified several candidate loci under this framework. However, the assumption of evolutionary independence is violated in case-control studies, where samples are drawn from the same population (Lotterhos and Whitlock 2014), such as when trypanosome-infected and uninfected flies from the same population are compared. More appropriate for this type of analysis is the haplotype-based analysis also used by Gloria-Soria *et al.* (2016), hapFLK (Fariello *et al.* 2013, 2014), because it corrects for group coancestry. Unfortunately, this approach lacked the resolution to identify outliers in the *Gff* data (Gloria-Soria *et al.* 2016).

Here, we applied genetic association methods, commonly used in human genetics and model organisms, to search for genetic determinants of trypanosome susceptibility in *Gff*. Association methods assume that common variants have modest effects on disease frequency and can explain the variability of a common disease [CD/CV hypothesis (Reich and Lander 2001)]. Their success in identifying a disease susceptibility locus relies on detecting increases in disease allele frequency in the affected individuals (Reich and Lander 2001). We applied a logistic regression of the phenotype on allele dosage that allows for multiple covariates, thus can control for sample stratification and coancestry by incorporating population structure into the analysis (Hill *et al.* 2017). LD-based clumping of the SNPs provided a parsimonious representative genome that greatly reduced the complexity of model fitting. Furthermore, this process allowed us to increase the strength of the association signal, which was otherwise hindered by background noise (Figure S1). We then performed a gene search that extended ≤ 2500 bp away from the selected representative SNPs. This was a conservative search space based on the average LD reported for these populations in Gloria-Soria *et al.* (2016). Longer LD regions are likely to occur around genes under strong selection and some genes may have been missed in this quest. This approach yielded 56 G*ff* candidate loci associated with trypanosome infection (Table S7), providing a good starting point to begin the functional validation. The search can be extended, as needed, for future analyses.

### Functional characterization of candidate genes

This study suggests that two main functional categories, cell proliferation and neurophysiology, may be important determinants of trypanosome infection outcomes in tsetse flies. Fifty-six genes were identified near SNPs statistically associated with trypanosome-parasitized individuals in *Gff*, and 29 of these genes were DE between *Trypanosoma*-infected and uninfected *Gmm* flies. Out of the 23 *Gff* genes with functional homologs in *Drosophila*, seven are involved in cell proliferation processes and DNA regulation (particularly in mitosis), and seven were associated with neurophysiology. These cell proliferation genes include *wge*, *pole2*, *msps*, *cdc14*, *moira*, *apc*, and *wee-1*. Midgut cell regeneration is an important process in the host defense mechanism against pathogens, and involves the delamination of damaged cells and their replacement with new ones derived from proliferating stem cells (Baton and Ranford-Cartwright 2007; Buchon *et al.* 2009a,b; Jiang *et al.* 2011). Differential expression of cell proliferation-associated genes has been observed between trypanosome-infected midgut and salivary gland epithelial tissues of tsetse flies (Aksoy *et al.* 2016; Telleria *et al.* 2014; Matetovici *et al.* 2016). The presence of the parasite within epithelial tissues likely disrupts cellular homeostasis, triggering the insect cell regeneration response. Since tissue integrity is essential for fighting invasive microbes (Buchon *et al.* 2009a), any mutation impairing cellular regeneration would have major consequences on the ability of tsetse flies to resist infectious trypanosomes. Among the seven genes assigned to the neurophysiology category, four gene products, homologous to *Drosophila ptp99A*, *malvolio*, *SV2B*, and an *insulin-like peptide* (Table 1), are DE in tsetse flies in response to infection. Little is known about the function of these genes in tsetse flies, but *Drosophila Ptp99A* is associated with microbiota-dependent nutriment functions (Dobson *et al.* 2015), and *insulin-like protein* belongs to a family of genes involved in feeding regulation (Pool and Scott 2014). Interestingly, *malvolio*, which is expressed in *Drosophila*’*s* central nervous system, macrophages, and midgut (Folwell *et al.* 2006), is required for normal taste behavior (Rodrigues *et al.* 1995). Any mechanism that may lead to an increase in blood-feeding frequency would concurrently increase the exposure of tsetse flies to parasites. Finally, among the three genes involved in the tsetse immune response are the *Drosophila* homologs of the *toll* and *dorsal* genes, which encode the receptor and transcription factors, respectively, of the Toll pathway. This immune pathway is a major component of the insect innate immune system (Valanne *et al.* 2011). In tsetse, the Toll pathway is induced upon parasite infection (Lehane *et al.* 2003) and activates the production of antimicrobial peptides that kill invasive microbes, including African trypanosomes (Boulanger *et al.* 2002). The identification of SNPs associated with trypanosome infection in two major genes of this pathway supports the important role of the insect immune response in the control of the parasite upon its entrance into the tsetse fly’s gut.

These findings are consistent with physiological outcomes associated with parasite infections in many insects, including tsetse flies, and encompass modifications in behavior and locomotion (Hurd 2003; van Houte *et al.* 2013; Caljon *et al.* 2016; Van Den Abbeele *et al.* 2010).

### Conclusions

The methodological approach used here allowed us to identify 56 trypanosome infection-associated loci in *Gff*, 29 of which were DE between *Trypanosoma*-infected and uninfected flies in the closely related species *Gmm*. The gene products identified by this screen are putatively involved in two main functional categories, cell proliferation and neurophysiology, suggesting that these processes may be determinants of the outcome of trypanosome infection in tsetse flies. Identification of the genetic determinants of trypanosome infection in *Gff*, and in tsetse flies in general, can provide targets for pharmaceutical and genetic interventions aimed at aiding the fight against the disease in endemic regions. Once disease-specific alleles have been identified, disease susceptibility could be actively monitored in the different tsetse populations by determining their frequency. This information can guide control efforts focused on disease elimination by prioritizing areas of higher risk. Future work will involve the screening of a large number of populations to both increase the sample size and cover different genetic backgrounds to validate this finding, followed by functional work on the top candidate genes identified by this work.

## Supplementary Material

Supplemental material is available online at www.g3journal.org/lookup/suppl/doi:10.1534/g3.117.300493/-/DC1.

## References

[bib1] Adolph S. K. DeLotto R. Nielsen F. C. Christiansen J., 2009 Embryonic expression of Drosophila IMP in the developing CNS and PNS. Gene Expr. Patterns 9: 138–143. 19111951 10.1016/j.gep.2008.12.001

[bib2] Aitchison J., 1982 The statistical analysis of compositional data. J. R. Stat. Soc. B 44: 139–177.

[bib3] Aksoy E. Telleria E. L. Echodu R. Wu Y. Okedi L. M., 2014 Analysis of multiple tsetse fly populations in Uganda reveals limited diversity and species-specific gut microbiota. Appl. Environ. Microbiol. 80: 4301–4312. 24814785 10.1128/AEM.00079-14 PMC4068677

[bib4] Aksoy E. Vigneron A. Bing X. Zhao X. O’Neill M., 2016 Mammalian African trypanosome VSG coat enhances tsetse’s vector competence. Proc. Natl. Acad. Sci. USA 113: 6961–6966. 27185908 10.1073/pnas.1600304113 PMC4922192

[bib5] Anders S. McCarthy D. J. Chen Y. Okoniewski M. Smyth G. K., 2013 Count-based differential expression analysis of RNA sequencing data using R and bioconductor. Nat. Protoc. 8: 1765–1786. 23975260 10.1038/nprot.2013.099

[bib6] Anders S. Pyl P. T. Huber W., 2015 HTSeq—a Python framework to work with high-throughput sequencing data. Bioinformatics 31: 166–169. 25260700 10.1093/bioinformatics/btu638 PMC4287950

[bib7] Andrews S., 2010 FastQC: a quality control tool for high throughput sequence data. Available at: http://www.bioinformatics.babraham.ac.uk/projects/fastqc. Accessed: January 29th, 2018.

[bib8] Attardo G. M. Benoit J. B. Michalkova V. Patrick K. R. Krause T. B., 2014 The homeodomain protein ladybird late regulates synthesis of milk proteins during pregnancy in the tsetse fly (Glossina morsitans). PLoS Negl. Trop. Dis. 8: e2645. 24763082 10.1371/journal.pntd.0002645 PMC3998940

[bib9] Baird N. A. Etter P. D. Atwood T. S. Currey M. C. Shiver A. L., 2008 Rapid SNP discovery and genetic mapping using sequenced RAD markers. PLoS One 3: e3376. 18852878 10.1371/journal.pone.0003376 PMC2557064

[bib10] Baton L. A. Ranford-Cartwright L. C., 2007 Morphological evidence for proliferative regeneration of the Anopheles stephensi midgut epithelium following Plasmodium falciparum ookinete invasion. J. Invertebr. Pathol. 96: 244–254. 17575986 10.1016/j.jip.2007.05.005

[bib11] Beadell J. S. Hyseni C. Abila P. P. Azabo R. Enyaru J. C. K., 2010 Phylogeography and population structure of Glossina fuscipes fuscipes in Uganda: implications for control of tsetse. PLoS Negl. Trop. Dis. 4: e636. 20300518 10.1371/journal.pntd.0000636 PMC2838784

[bib12] Beaumont M. A., 2005 Adaptation and speciation: what can F ST tell us? Trends Ecol. Evol. 20: 435–440. 16701414 10.1016/j.tree.2005.05.017

[bib13] Benjamini Y. Hochberg Y., 1995 Controlling the false discovery rate: a practical and powerful approach to multiple testing. J. R. Stat. Soc. B Stat. Methodol. 57: 289–300.

[bib14] Benoit J. B. Vigneron A. Broderick N. A. Wu Y. Sun J. S., 2017 Symbiont-induced odorant binding proteins mediate insect host hematopoiesis. Elife 6: e19535. 28079523 10.7554/eLife.19535 PMC5231409

[bib15] Berg J. J. Coop G., 2014 A population genetic signal of polygenic adaptation. PLoS Genet. 10: e1004412. 25102153 10.1371/journal.pgen.1004412 PMC4125079

[bib16] Boulanger N. Brun R. Ehret-Sabatier L. Kunz C. Bulet P., 2002 Immunopeptides in the defense reactions of Glossina morsitans to bacterial and Trypanosoma brucei brucei infections. Insect Biochem. Mol. Biol. 32: 369–375. 11886771 10.1016/s0965-1748(02)00029-2

[bib17] Brelsfoard C. Tsiamis G. Falchetto M. Gomulski L. M. Telleria E., 2014 Presence of extensive Wolbachia symbiont insertions discovered in the genome of its host Glossina morsitans morsitans. PLoS Negl. Trop. Dis. 8: e2728. 24763283 10.1371/journal.pntd.0002728 PMC3998919

[bib18] Buchon N. Broderick N. A. Chakrabarti S. Lemaitre B., 2009 a Invasive and indigenous microbiota impact intestinal stem cell activity through multiple pathways in Drosophila. Genes Dev. 23: 2333–2344. 19797770 10.1101/gad.1827009 PMC2758745

[bib19] Buchon N. Broderick N. A. Poidevin M. Pradervand S. Lemaitre B., 2009 b Drosophila intestinal response to bacterial infection: activation of host defense and stem cell proliferation. Cell Host Microbe 5: 200–211. 19218090 10.1016/j.chom.2009.01.003

[bib20] Caljon G. De Muylder G. Durnez L. Jennes W. Vanaerschot M., 2016 Alice in microbes’ land: adaptations and counter-adaptations of vector-borne parasitic protozoa and their hosts. FEMS Microbiol. Rev. 40: 664–685. 27400870 10.1093/femsre/fuw018

[bib21] Cardoso G. A. Marinho M. A. T. Monfardini R. D. de Azeredo Espin A. M. L. Torres T. T., 2016 Evolution of genes involved in feeding preference and metabolic processes in Calliphoridae (Diptera: Calyptratae). PeerJ 4: e2598. 27812410 10.7717/peerj.2598 PMC5088637

[bib22] Chang C. C. Chow C. C. Tellier L. C. A. M. Vattikuti S. Purcell S. M., 2015 Second-generation PLINK: rising to the challenge of larger and richer datasets. Gigascience 4: 7. 25722852 10.1186/s13742-015-0047-8 PMC4342193

[bib23] Crosby M. A. Miller C. Alon T. Watson K. L. Verrijzer C. P., 1999 The trithorax group gene moira encodes a brahma-associated putative chromatin-remodeling factor in Drosophila melanogaster. Mol. Cell. Biol. 19: 1159–1170. 9891050 10.1128/mcb.19.2.1159 PMC116045

[bib24] Cullen C. F. Deak P. Glover D. M. Ohkura H., 1999 mini spindles: a gene encoding a conserved microtubule-associated protein required for the integrity of the mitotic spindle in Drosophila. J. Cell Biol. 146: 1005–1018. 10477755 10.1083/jcb.146.5.1005 PMC2169485

[bib25] David J. P. Faucon F. Chandor-Proust A. Poupardin R. Riaz M. A., 2014 Comparative analysis of response to selection with three insecticides in the dengue mosquito Aedes aegypti using mRNA sequencing. BMC Genomics 15: 174. 24593293 10.1186/1471-2164-15-174 PMC4029067

[bib26] Desai C. J. Krueger N. X. Saito H. Zinn K., 1997 Competition and cooperation among receptor tyrosine phosphatases control motoneuron growth cone guidance in Drosophila. Development 124: 1941–1952. 9169841 10.1242/dev.124.10.1941

[bib27] Dobson A. J. Chaston J. M. Newell P. D. Donahue L. Hermann S. L., 2015 Host genetic determinants of microbiota-dependent nutrition revealed by genome-wide analysis of Drosophila melanogaster. Nat. Commun. 6: 6312. 25692519 10.1038/ncomms7312 PMC4333721

[bib28] Duforet-Frebourg N. Bazin E. Blum M. G., 2014 Genome scans for detecting footprints of local adaptation using a Bayesian factor model. Mol. Biol. Evol. 31: 2483–2495. 24899666 10.1093/molbev/msu182 PMC4137708

[bib29] Echodu R. Beadell J. S. Okedi L. M. Hyseni C. Aksoy S., 2011 Temporal stability of Glossina fuscipes fuscipes populations in Uganda. Parasit. Vectors 4: 19. 21320301 10.1186/1756-3305-4-19 PMC3045980

[bib30] Enright A. J. John B. Gaul U. Tuschl T. Sander C., 2003 MicroRNA targets in Drosophila. Genome Biol. 5: R1. 14709173 10.1186/gb-2003-5-1-r1 PMC395733

[bib31] Fariello M. I. Boitard S. Naya H. SanCristobal M. Servin B., 2013 Detecting signatures of selection through haplotype differentiation among hierarchically structured populations. Genetics 193: 929–941. 23307896 10.1534/genetics.112.147231 PMC3584007

[bib32] Fariello M.-I. Servin B. Tosser-Klopp G. Rupp R. Moreno C., 2014 Selection signatures in worldwide sheep populations. PLoS One 9: e103813. 25126940 10.1371/journal.pone.0103813 PMC4134316

[bib33] Finn R. D. Coggill P. Eberhardt R. Y. Eddy S. R. Mistry J., 2016 The Pfam protein families database: towards a more sustainable future. Nucleic Acids Res. 44: D279–D285. 26673716 10.1093/nar/gkv1344 PMC4702930

[bib34] Fischer M. C. Foll M. Excoffier L. Heckel G., 2011 Enhanced AFLP genome scans detect local adaptation in high-altitude populations of a small rodent (Microtus arvalis). Mol. Ecol. 20: 1450–1462. 21352386 10.1111/j.1365-294X.2011.05015.x

[bib35] Folwell J. L. Barton C. H. Shepherd D., 2006 Immunolocalisation of the D. melanogaster Nramp homologue Malvolio to gut and Malpighian tubules provides evidence that Malvolio and Nramp2 are orthologous. J. Exp. Biol. 209: 1988–1995. 16651563 10.1242/jeb.02193

[bib36] Garelli A. Gontijo A. M. Miguela V. Caparros E. Dominguez M., 2012 Imaginal discs secrete insulin-like peptide 8 to mediate plasticity of growth and maturation. Science 336: 579–582. 22556250 10.1126/science.1216735

[bib37] Geiger A. Hamidou Soumana I. Tchicaya B. Rofidal V. Decourcelle M., 2015 Differential expression of midgut proteins in Trypanosoma brucei gambiense-stimulated *vs.* non-stimulated Glossina palpalis gambiensis flies. Front. Microbiol. 6: 444. 26029185 10.3389/fmicb.2015.00444 PMC4428205

[bib38] Giraldo-Calderón G. I. Emrich S. J. MacCallum R. M. Maslen G. Dialynas E., 2015 VectorBase: an updated bioinformatics resource for invertebrate vectors and other organisms related with human diseases. Nucleic Acids Res. 43: D707–D713. 25510499 10.1093/nar/gku1117 PMC4383932

[bib39] Gloria-Soria A. Dunn W. A. Telleria E. L. Evans B. R. Okedi L., 2016 Patterns of genome-wide variation in Glossina fuscipes fuscipes tsetse flies from Uganda. G3 (Bethesda) 6: 1573–1584. 27172181 10.1534/g3.116.027235 PMC4889654

[bib40] Gramates L. S. Marygold S. J. dos Santos G. Urbano J.-M. Antonazzo G., 2017 FlyBase at 25: looking to the future. Nucleic Acids Res. 45: D663–D671. 27799470 10.1093/nar/gkw1016 PMC5210523

[bib41] Hamidou Soumana I. Tchicaya B. Rialle S. Parrinello H. Geiger A., 2017 Comparative genomics of Glossina palpalis gambiensis and G. morsitans morsitans to reveal gene orthologs involved in infection by Trypanosoma brucei gambiense. Front. Microbiol. 8: 540. 28421044 10.3389/fmicb.2017.00540 PMC5376623

[bib42] Harris C. Rousset F. Morlais I. Fontenille D. Cohuet A., 2010 Low linkage disequilibrium in wild Anopheles gambiae s.l. populations. BMC Genet. 11: 81. 20843306 10.1186/1471-2156-11-81 PMC2949739

[bib43] Hill A. Loh P.-R. Bharadwaj R. B. Pons P. Shang J., 2017 Stepwise distributed open innovation contests for software development: acceleration of genome-wide association analysis. Gigascience 6: 1–10. 10.1093/gigascience/gix009 PMC5467032 28327993

[bib44] Hirota Y. Sawamoto K. Takahashi K. Ueda R. Okano H., 2005 The transmembrane protein, Tincar, is involved in the development of the compound eye in Drosophila melanogaster. Dev. Genes Evol. 215: 90–96. 15654626 10.1007/s00427-004-0452-y

[bib45] Hurd H., 2003 Manipulation of medically important insect vectors by their parasites. Annu. Rev. Entomol. 48: 141–161. 12414739 10.1146/annurev.ento.48.091801.112722

[bib46] Hyseni C. Kato A. B. Okedi L. M. Masembe C. Ouma J. O., 2012 The population structure of Glossina fuscipes fuscipes in the Lake Victoria basin in Uganda: implications for vector control. Parasit. Vectors 5: 222. 23036153 10.1186/1756-3305-5-222 PMC3522534

[bib47] Jensen J. D. Foll M. Bernatchez L., 2016 The past, present and future of genomic scans for selection. Mol. Ecol. 25: 1–4. 26745554 10.1111/mec.13493

[bib48] Jiang H. Grenley M. O. Bravo M.-J. Blumhagen R. Z. Edgar B. A., 2011 EGFR/Ras/MAPK signaling mediates adult midgut epithelial homeostasis and regeneration in Drosophila. Cell Stem Cell 8: 84–95. 21167805 10.1016/j.stem.2010.11.026 PMC3021119

[bib49] Kent W. J., 2002 BLAT--the BLAST-like alignment tool. Genome Res. 12: 656–664. 11932250 10.1101/gr.229202 PMC187518

[bib50] Kim D. Pertea G. Trapnell C. Pimentel H. Kelley R., 2013 TopHat2: accurate alignment of transcriptomes in the presence of insertions, deletions and gene fusions. Genome Biol. 14: R36. 23618408 10.1186/gb-2013-14-4-r36 PMC4053844

[bib51] Kunttas-Tatli E. Von Kleeck R. A. Greaves B. D. Vinson D. Roberts D. M., 2015 The two SAMP repeats and their phosphorylation state in Drosophila Adenomatous polyposis coli-2 play mechanistically distinct roles in negatively regulating Wnt signaling. Mol. Biol. Cell 26: 4503–4518. 26446838 10.1091/mbc.E15-07-0515 PMC4666143

[bib52] Langmead B. Trapnell C. Pop M. Salzberg S. L., 2009 Ultrafast and memory-efficient alignment of short DNA sequences to the human genome. Genome Biol. 10: R25. 19261174 10.1186/gb-2009-10-3-r25 PMC2690996

[bib53] Lee W. C. Beebe K. Sudmeier L. Micchelli C. A., 2009 Adenomatous polyposis coli regulates Drosophila intestinal stem cell proliferation. Development 136: 2255–2264. 19502486 10.1242/dev.035196 PMC13471719

[bib54] Lehane M. J. Aksoy S. Gibson W. Kerhornou A. Berriman M., 2003 Adult midgut expressed sequence tags from the tsetse fly Glossina morsitans morsitans and expression analysis of putative immune response genes. Genome Biol. 4: R63. 14519198 10.1186/gb-2003-4-10-r63 PMC328452

[bib55] Lemaitre B. Nicolas E. Michaut L. Reichhart J. M. Hoffmann J. A., 1996 The dorsoventral regulatory gene cassette spatzle/Toll/cactus controls the potent antifungal response in Drosophila adults. Cell 86: 973–983. 8808632 10.1016/s0092-8674(00)80172-5

[bib56] Liu Q. Yang X. Tian J. Gao Z. Wang M., 2016 Gap junction networks in mushroom bodies participate in visual learning and memory in Drosophila. Elife 5: e13238. 10.7554/eLife.13238 PMC4909397 27218450

[bib57] Lotterhos K. E. Whitlock M. C., 2014 Evaluation of demographic history and neutral parameterization on the performance of FST outlier tests. Mol. Ecol. 23: 2178–2192. 24655127 10.1111/mec.12725 PMC4228763

[bib58] Mackay T. F. Richards S. Stone E. A. Barbadilla A. Ayroles J. F., 2012 The Drosophila melanogaster genetic reference panel. Nature 482: 173–178. 22318601 10.1038/nature10811 PMC3683990

[bib59] Mariette S. Wong Jun Tai F. Roch G. Barre A. Chague A., 2016 Genome‐wide association links candidate genes to resistance to Plum Pox virus in apricot (Prunus armeniaca). New Phytol. 209: 773–784. 26356603 10.1111/nph.13627

[bib60] Marsden C. D. Lee Y. Kreppel K. Weakley A. Cornel A., 2014 Diversity, differentiation, and linkage disequilibrium: prospects for association mapping in the malaria vector Anopheles arabiensis. G3 (Bethesda) 4: 121–131. 24281424 10.1534/g3.113.008326 PMC3887528

[bib61] Matetovici I. Caljon G. Van Den Abbeele J., 2016 Tsetse fly tolerance to T. brucei infection: transcriptome analysis of trypanosome-associated changes in the tsetse fly salivary gland. BMC Genomics 17: 971. 27884110 10.1186/s12864-016-3283-0 PMC5123318

[bib62] Medioni C. Ramialison M. Ephrussi A. Besse F., 2014 Imp promotes axonal remodeling by regulating profilin mRNA during brain development. Curr. Biol. 24: 793–800. 24656828 10.1016/j.cub.2014.02.038

[bib63] Morgans C. W. Kensel-Hammes P. Hurley J. B. Burton K. Idzerda R., 2009 Loss of the synaptic vesicle protein SV2B results in reduced neurotransmission and altered synaptic vesicle protein expression in the retina. PLoS One 4: e5230. 19381277 10.1371/journal.pone.0005230 PMC2667261

[bib64] Munks R. J. Sant’Anna M. R. Grail W. Gibson W. Igglesden T., 2005 Antioxidant gene expression in the blood-feeding fly Glossina morsitans morsitans. Insect Mol. Biol. 14: 483–491. 16164604 10.1111/j.1365-2583.2005.00579.x

[bib65] Nakamura K. Ida H. Yamaguchi M., 2008 Transcriptional regulation of the Drosophila moira and osa genes by the DREF pathway. Nucleic Acids Res. 36: 3905–3915. 18511465 10.1093/nar/gkn291 PMC2475616

[bib66] Narum S. R. Hess J. E., 2011 Comparison of F(ST) outlier tests for SNP loci under selection. Mol. Ecol. Resour. 11: 184–194. 21429174 10.1111/j.1755-0998.2011.02987.x

[bib67] Opiro R. Saarman N. P. Echodu R. Opiyo E. A. Dion K., 2016 Evidence of temporal stability in allelic and mitochondrial haplotype diversity in populations of Glossina fuscipes fuscipes (Diptera: Glossinidae) in northern Uganda. Parasit. Vectors 9: 258. 27141947 10.1186/s13071-016-1522-5 PMC4855780

[bib68] Opiro R. Saarman N. P. Echodu R. Opiyo E. A. Dion K., 2017 Genetic diversity and population structure of the tsetse fly Glossina fuscipes fuscipes (Diptera: Glossinidae) in Northern Uganda: implications for vector control. PLoS Negl. Trop. Dis. 11: e0005485. 28453513 10.1371/journal.pntd.0005485 PMC5425221

[bib69] Ozawa N. Furuhashi H. Masuko K. Numao E. Makino T., 2016 Organ identity specification factor WGE localizes to the histone locus body and regulates histone expression to ensure genomic stability in Drosophila. Genes Cells 21: 442–456. 27145109 10.1111/gtc.12354

[bib70] Petersen F. T. Meier R. Kutty S. N. Wiegmann B. M., 2007 The phylogeny and evolution of host choice in the Hippoboscoidea (Diptera) as reconstructed using four molecular markers. Mol. Phylogenet. Evol. 45: 111–122. 17583536 10.1016/j.ympev.2007.04.023

[bib71] Peterson B. K. Weber J. N. Kay E. H. Fisher H. S. Hoekstra H. E., 2012 Double digest RADseq: an inexpensive method for de novo SNP discovery and genotyping in model and non-model species. PLoS One 7: e37135. 22675423 10.1371/journal.pone.0037135 PMC3365034

[bib72] Picozzi K. Fèvre E. Odiit M. Carrington M. Eisler M. C., 2005 Sleeping sickness in Uganda: a thin line between two fatal diseases. BMJ 331: 1238–1241. 16308383 10.1136/bmj.331.7527.1238 PMC1289320

[bib73] Pool A.-H. Scott K., 2014 Feeding regulation in Drosophila. Curr. Opin. Neurobiol. 29: 57–63. 24937262 10.1016/j.conb.2014.05.008 PMC4253568

[bib74] Purcell S. Neale B. Todd-Brown K. Thomas L. Ferreira M. A., 2007 PLINK: a tool set for whole-genome association and population-based linkage analyses. Am. J. Hum. Genet. 81: 559–575. 17701901 10.1086/519795 PMC1950838

[bib75] Raj A. Stephens M. Pritchard J. K., 2014 fastSTRUCTURE: variational inference of population structure in large SNP data sets. Genetics 197: 573–589. 24700103 10.1534/genetics.114.164350 PMC4063916

[bib76] Reich D. E. Lander E. S., 2001 On the allelic spectrum of human disease. Trends Genet. 17: 502–510. 11525833 10.1016/s0168-9525(01)02410-6

[bib77] Rio R. V. M. Symula R. E. Wang J. Lohs C. Wu Y., 2012 Insight into the transmission biology and species-specific functional capabilities of tsetse (Diptera: glossinidae) obligate symbiont Wigglesworthia. MBio 3: e00240-11. 22334516 10.1128/mBio.00240-11 PMC3280448

[bib78] Risso D. Ngai J. Speed T. P. Dudoit S., 2014 Normalization of RNA-seq data using factor analysis of control genes or samples. Nat. Biotechnol. 32: 896–902. 25150836 10.1038/nbt.2931 PMC4404308

[bib79] Roberts A. Pimentel H. Trapnell C. Pachter L., 2011 a Identification of novel transcripts in annotated genomes using RNA-Seq. Bioinformatics 27: 2325–2329. 21697122 10.1093/bioinformatics/btr355

[bib80] Roberts A. Trapnell C. Donaghey J. Rinn J. L. Pachter L., 2011 b Improving RNA-Seq expression estimates by correcting for fragment bias. Genome Biol. 12: R22. 21410973 10.1186/gb-2011-12-3-r22 PMC3129672

[bib81] Robinson M. D. Oshlack A., 2010 A scaling normalization method for differential expression analysis of RNA-seq data. Genome Biol. 11: R25. 20196867 10.1186/gb-2010-11-3-r25 PMC2864565

[bib82] Robinson M. D. Smyth G. K., 2007 Moderated statistical tests for assessing differences in tag abundance. Bioinformatics 23: 2881–2887. 17881408 10.1093/bioinformatics/btm453

[bib83] Robinson M. D. Smyth G. K., 2008 Small-sample estimation of negative binomial dispersion, with applications to SAGE data. Biostatistics 9: 321–332. 17728317 10.1093/biostatistics/kxm030

[bib84] Robinson M. D. McCarthy D. J. Smyth G. K., 2010 edgeR: a bioconductor package for differential expression analysis of digital gene expression data. Bioinformatics 26: 139–140. 19910308 10.1093/bioinformatics/btp616 PMC2796818

[bib85] Robinson M. D. Strbenac D. Stirzaker C. Statham A. L. Song J. Z., 2012 Copy-number-aware differential analysis of quantitative DNA sequencing data. Genome Res. 22: 2489–2496. 22879430 10.1101/gr.139055.112 PMC3514678

[bib86] Rodrigues V. Cheah P. Y. Ray K. Chia W., 1995 malvolio, the Drosophila homologue of mouse NRAMP-1 (Bcg), is expressed in macrophages and in the nervous system and is required for normal taste behaviour. EMBO J. 14: 3007. 7621816 10.1002/j.1460-2075.1995.tb07303.x PMC394361

[bib87] Sahashi R. Matsuda R. Suyari O. Kawai M. Yoshida H., 2013 Functional analysis of Drosophila DNA polymerase epsilon p58 subunit. Am. J. Cancer Res. 3: 478–489. 24224125 PMC3816967

[bib88] Schoville S. D. Bonin A. François O. Lobreaux S. Melodelima C., 2012 Adaptive genetic variation on the landscape: methods and cases. Annu. Rev. Ecol. Evol. Syst. 43: 23–43.

[bib89] Stegmeier F. Amon A., 2004 Closing mitosis: the functions of the Cdc14 phosphatase and its regulation. Annu. Rev. Genet. 38: 203–232. 15568976 10.1146/annurev.genet.38.072902.093051

[bib90] Stumpff J. Kellogg D. R. Krohne K. A. Su T. T., 2005 Drosophila Wee1 interacts with members of the gammaTURC and is required for proper mitotic-spindle morphogenesis and positioning. Curr. Biol. 15: 1525–1534. 16139207 10.1016/j.cub.2005.07.031 PMC3242738

[bib91] Telleria E. L. Benoit J. B. Zhao X. Savage A. F. Regmi S., 2014 Insights into the trypanosome-host interactions revealed through transcriptomic analysis of parasitized tsetse fly salivary glands. PLoS Negl. Trop. Dis. 8: e2649. 24763140 10.1371/journal.pntd.0002649 PMC3998935

[bib92] Thompson E. C. Travers A. A., 2008 A Drosophila Smyd4 homologue is a muscle-specific transcriptional modulator involved in development. PLoS One 3: e3008. 18714374 10.1371/journal.pone.0003008 PMC2500188

[bib93] Toh H. Weiss B. L. Perkin S. A. H. Yamashita A. Oshima K., 2006 Massive genome erosion and functional adaptations provide insights into the symbiotic lifestyle of Sodalis glossinidius in the tsetse host. Genome Res. 16: 149–156. 16365377 10.1101/gr.4106106 PMC1361709

[bib94] Trapnell C. Roberts A. Goff L. Pertea G. Kim D., 2012 Differential gene and transcript expression analysis of RNA-seq experiments with TopHat and Cufflinks. Nat. Protoc. 7: 562–578. 22383036 10.1038/nprot.2012.016 PMC3334321

[bib95] Valanne S. Wang J. H. Ramet M., 2011 The Drosophila Toll signaling pathway. J. Immunol. 186: 649–656. 21209287 10.4049/jimmunol.1002302

[bib96] Van Den Abbeele J. Caljon G. De Ridder K. De Baetselier P. Coosemans M., 2010 Trypanosoma brucei modifies the tsetse salivary composition, altering the fly feeding behavior that favors parasite transmission. PLoS Pathog. 6: e1000926. 20532213 10.1371/journal.ppat.1000926 PMC2880569

[bib97] van Houte S. Ros V. I. van M. M. Oers, 2013 Walking with insects: molecular mechanisms behind parasitic manipulation of host behaviour. Mol. Ecol. 22: 3458–3475. 23742168 10.1111/mec.12307

[bib98] Walmsley S. J. Gaines P. J., 2004 Identification of two cDNAs encoding synaptic vesicle protein 2 (SV2)‐like proteins from epithelial tissues in the cat flea, Ctenocephalides felis. Insect Mol. Biol. 13: 225–230. 15157223 10.1111/j.0962-1075.2004.00478.x

[bib99] Welburn S. C. Maudlin I. Simarro P. P., 2009 Controlling sleeping sickness–a review. Parasitology 136: 1943–1949. 19691861 10.1017/S0031182009006416

