# Supplementary material for: Uncovering Genomic Regions Associated with Trypanosoma Infections in Wild Populations of the Tsetse Fly Glossina fuscipes
Source: G3 (Bethesda). 2018 Jan 17;8(3):887–97. doi: 10.1534/g3.117.300493 (PMC5844309; doi:10.1534/g3.117.300493)
Supplement: Supplementary file 1 [file 887FigureS1.pdf]

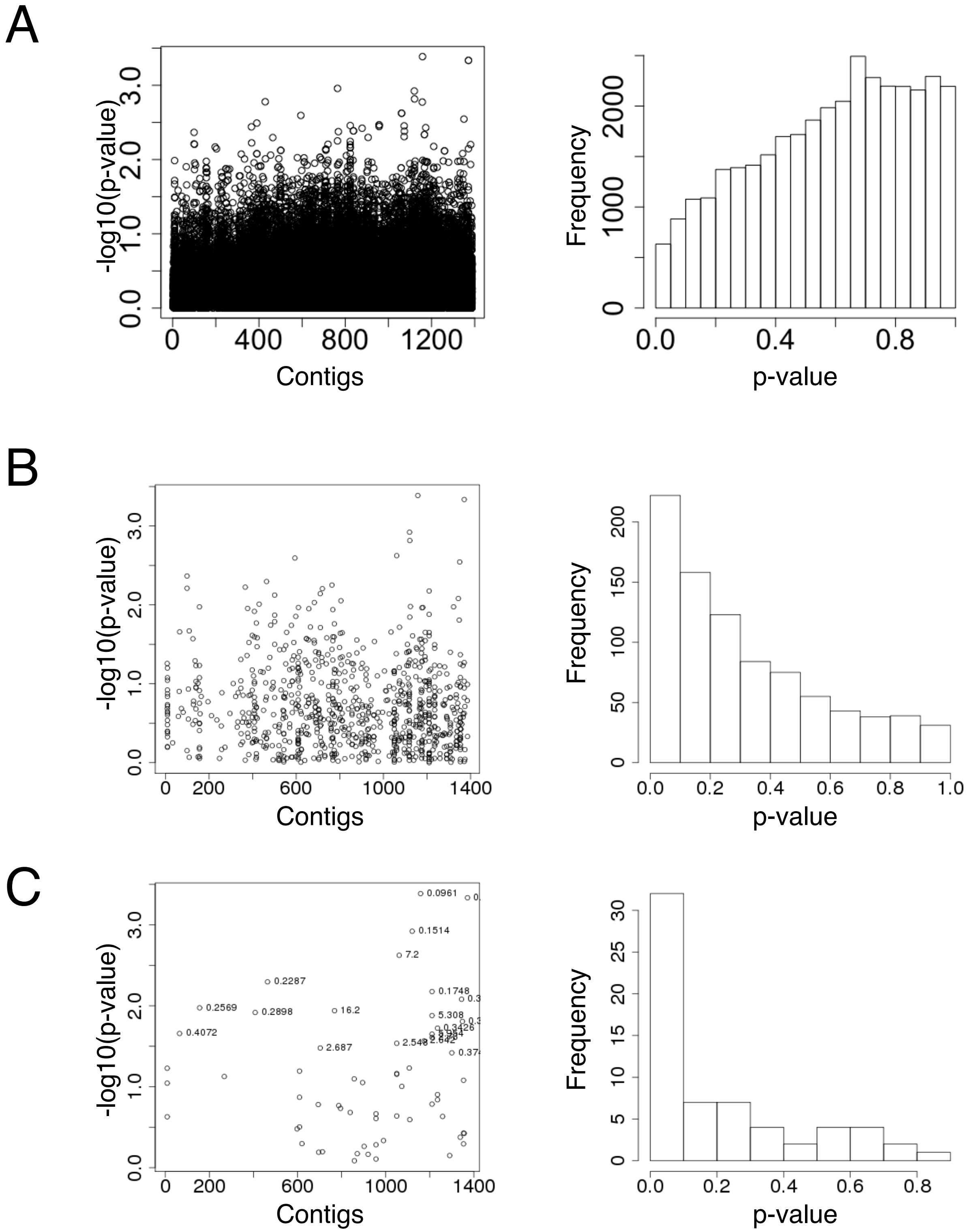

Figure S1: Manhattan plot before (A) and after (B and C) the LD pruning, and the corresponding histograms of p-values for different LD threshold = 10% (B) and 1% (C).
The numbers above dots are the estimated odds ratios for significant SNPs (p-value<.05).
