# Supplementary material for: Uncovering Genomic Regions Associated with Trypanosoma Infections in Wild Populations of the Tsetse Fly Glossina fuscipes
Source: G3 (Bethesda). 2018 Jan 17;8(3):887–97. doi: 10.1534/g3.117.300493 (PMC5844309; doi:10.1534/g3.117.300493)
Supplement: Supplementary file 2 [file 887FigureS2.pdf]

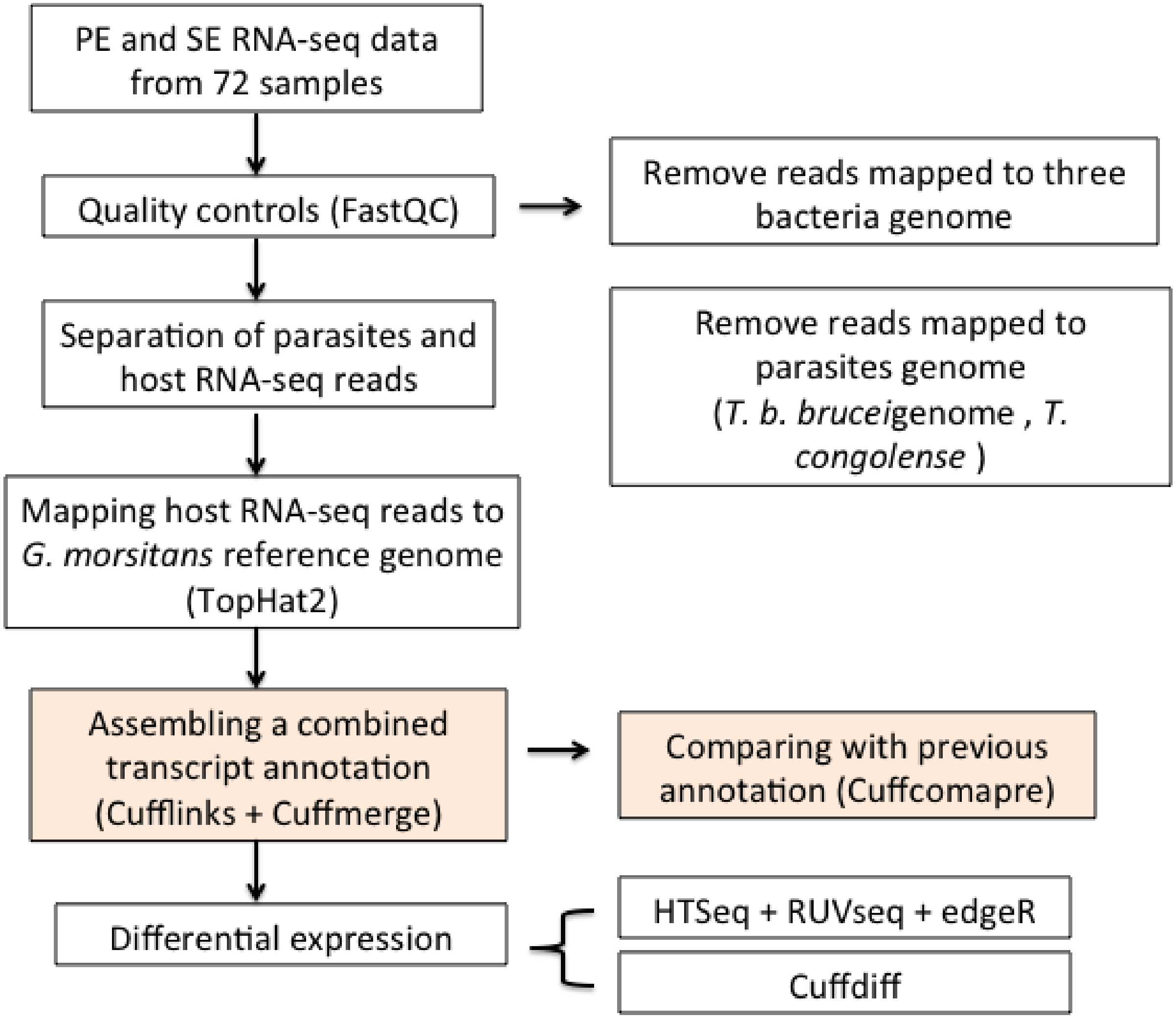

Figure S2: Analysis pipeline of the Glossina morsitans transcriptome analysis of the host, bacteria and parasite RNA-seq reads from 72 Glossina morsitans samples. Cufflinks, Cuffmerge, Cuffcompare, and Cuffdiff are programs included in the Cufflinks suite.
