# Supplementary material for: Uncovering Genomic Regions Associated with Trypanosoma Infections in Wild Populations of the Tsetse Fly Glossina fuscipes
Source: G3 (Bethesda). 2018 Jan 17;8(3):887–97. doi: 10.1534/g3.117.300493 (PMC5844309; doi:10.1534/g3.117.300493)
Supplement: Supplementary file 3 [file 887FigureS3.pdf]

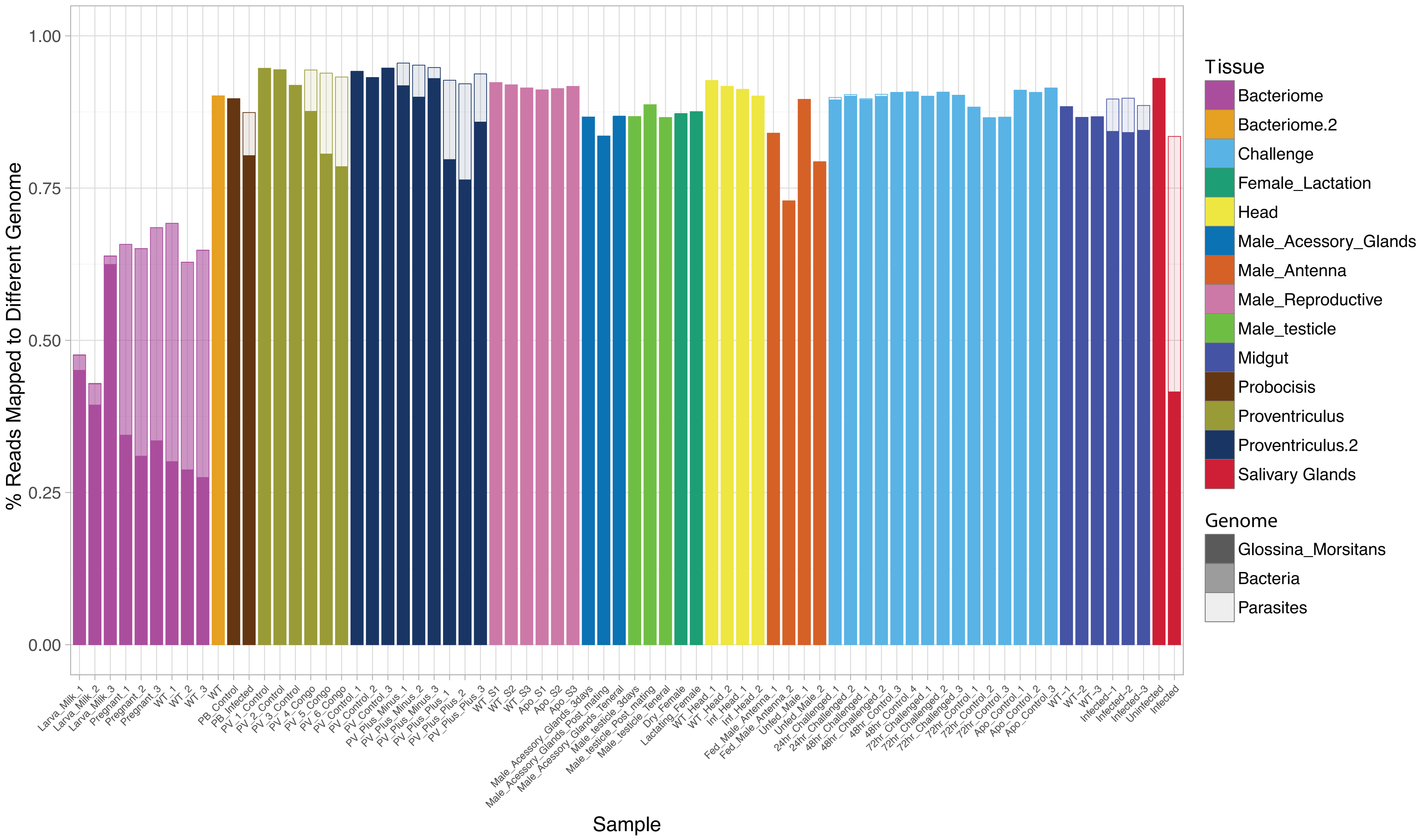

Figure S3: Percentage of reads aligned to the host, bacteria, and parasites genome in the 72 Glossina morsitans samples. Tissue types were coded with different colors; reads aligned to the host, bacteria, and parasites genomes were represented by dark, median, and light shades of color, respectively.
