# Supplementary material for: Uncovering Genomic Regions Associated with Trypanosoma Infections in Wild Populations of the Tsetse Fly Glossina fuscipes
Source: G3 (Bethesda). 2018 Jan 17;8(3):887–97. doi: 10.1534/g3.117.300493 (PMC5844309; doi:10.1534/g3.117.300493)
Supplement: Supplementary file 5 [file 887TableS1.docx]

**Table S1:** Summary of the 72 transcriptomes used in the transcriptomic analysis.

| **Tissue** | **Sex** | **Stage** | **Age** | **Attribute** | **Parasite** | **Rep** | **Sequences accession** | **Reference** |
| --- | --- | --- | --- | --- | --- | --- | --- | --- |
| Cardia | F | A | 40D | Wild type | N/A | 3 | SRR5124386, SRR5124388, SRR5124389 | N/A |
| Cardia | F | A | 40D | Infected (midgut only) | Tbb | 3 | SRR5124390, SRR5124391, SRR5124392 | N/A |
| Cardia | F | A | 40D | Infected (midgut and salivary glands) | Tbb | 3 | SRR5124387, SRR5124385, SRR5124393 | N/A |
| Salivary glands | F | A | 45D | Wild type | N/A | 1 | SRR965340 | Telleria et al., 2014 |
| Salivary glands | F | A | 45D | Infected (midgut and salivary glands) | Tbb | 1 | SRR965341 | Telleria et al., 2014 |
| Testes | M | A | Teneral | Wild type | N/A | 1 | SRX1254417 | Scolari et al., 2016 |
| Testes | M | A | 3D | Wild type | N/A | 1 | SRX1254425 | Scolari et al., 2016 |
| Testes | M | A | 3.5D | Wild type | N/A | 1 | SRX1254426 | Scolari et al., 2016 |
| Accessory Glands | M | A | Teneral | Wild type | N/A | 1 | SRX1251080 | Scolari et al., 2016 |
| Accessory Glands | M | A | 3D | Wild type | N/A | 1 | SRX1251081 | Scolari et al., 2016 |
| Accessory Glands | M | A | 3.5D | Wild type | N/A | 1 | SRX1254054 | Scolari et al., 2016 |
| Whole body | F | A | 16-18D | Non lactating (post parturition) | N/A | 1 | SRX287395 | Benoit et al., 2014 |
| Whole body | F | A | 16-18D | Lactating (3rd instar larvae) | N/A | 1 | SRX287393 | Benoit et al., 2014 |
| Whole midgut | F | A | 9D | Challenged 24h | Tbr | 2 | PRJNA314786 | Aksoy et al., 2016 |
| Whole midgut | F | A | 10D | Challenged 48h | Tbr | 2 | PRJNA314786 | Aksoy et al., 2016 |
| Whole midgut | F | A | 11D | Challenged 72h | Tbr | 3 | PRJNA314786 | Aksoy et al., 2016 |
| Whole midgut | F | A | 10D | Wild type | N/A | 2 | PRJNA314786 | Aksoy et al., 2016 |
| Whole midgut | F | A | 11D | Wild type | N/A | 3 | PRJNA314786 | Aksoy et al., 2016 |
| Whole midgut | F | A | 11D | Aposymbiotic | N/A | 3 | SRR5207250-2 | Bing, Attardo et al., 2017 |
| Whole midgut | F | A | 20D | Wild type | N/A | 3 | PRJNA314786 | N/A |
| Whole midgut | F | A | 20D | Infected (midgut only) | Tbr | 3 | PRJNA314786 | N/A |
| Proboscis | F | A | 28D | Wild type | N/A | 1 | SRP093552 | Awuoche et al., 2017 |
| Proboscis | F | A | 28D | Infected | Tc | 1 | SRP093552 | Awuoche et al., 2017 |
| Cardia | F | A | 28D | Wild type | N/A | 3 | SRP093558 | N/A |
| Cardia | F | A | 28D | Infected | Tc | 3 | SRP093558 | N/A |
| Bacteriome | F | A | 16-18D | Virgin | N/A | 3 | PRJNA335358 | Bing, Attardo et al., 2017 |
| Bacteriome | F | A | 16-18D | Pregnant (2nd instar) | N/A | 3 | PRJNA335358 | Bing, Attardo et al., 2017 |
| Bacteriome | F | A | 14D | Wild type | N/A | 1 | PRJNA335358 | N/A |
| Testes and accessory glands | M | A | 7D | Wild type | N/A | 3 | PRJNA394896 | N/A |
| Testes and accessory glands | M | A | 7D | Aposymbiotic | N/A | 3 | PRJNA394896 | N/A |
| Milk | F | L | 2nd instar | Wild type | N/A | 3 | PRJNA429038 | N/A |
| Head | M | A | Adult | Wild type | N/A | 2 | SRP090041 | Awuoche et al., 2017 |
| Head | M | A | Adult | Infected |  | 2 | SRP090041 | Awuoche et al., 2017 |
| Antenna | M | A | Adult | Unfed | N/A | 2 | PRJNA429025 | N/A |
| Antenna | M | A | Adult | Fed | N/A | 2 | PRJNA429025 | N/A |

Note: Listed are the tissues from which the transcriptomic data originated and their respective features, including: sex of flies (F=female; M=male), flies developmental stage (A=adult; L=larva), the age of flies (D=days), the type of parasite when flies were infected (Tbb=T. brucei brucei RUMP 503; Tbr= *T.brucei rhodesiense* YTAT; Tc= *T. congolense*), the number of replicates for each transcriptome, the SRA accession numbers, and references for these transcriptomes when previously published.

**Associated References:**

Aksoy, E., A. Vigneron, X. Bing, X. Zhao, M. O’Neill et al., 2016 Mammalian African trypanosome VSG coat enhances tsetse’s vector competence. Proc. Natl. Acad. Sci. 201600304.

Awuoche, E.O., Weiss, B.L., Vigneron, A., Mireji, P.O., Aksoy, E., Nyambega, B., Attardo, G.M., Wu, Y., O'Neill, M., Murilla, G., et al. 2017 Molecular characterization of tsetse's proboscis and its response to Trypanosoma congolense infection. PLOS Negl Trop Dis 11, e0006057. (doi:10.1371/journal.pntd.0006057).

Bing, X., Attardo, G.M., Vigneron, A., Aksoy, E., Scolari, F., Malacrida, A., Weiss, B.L. & Aksoy, S. 2017 Unravelling the relationship between the tsetse fly and its obligate symbiont Wigglesworthia: transcriptomic and metabolomic landscapes reveal highly integrated physiological networks. Proc Biol Sci 284. (doi:10.1098/rspb.2017.0360).

Benoit, J.B., Vigneron, A., Broderick, N.A., Wu, Y., Sun, J.S., Carlson, J.R., Aksoy, S. and Weiss, B.L., 2017. Symbiont-induced odorant binding proteins mediate insect host hematopoiesis. Elife, 6, p.e19535.

Scolari F., Benoit J.B., Michalkova V., Aksoy E., Takac P., Abd-Alla A.M.M., Malacrida A.R., Aksoy S., and Attardo G.M. 2016 The spermatophore in *Glossina morsitans morsitans*: Insights into male contributions to reproduction. Scientific Reports 6: 20334.

Telleria, E. L., J. B. Benoit, X. Zhao, A. F. Savage, S. Regmi et al., 2014 Insights into the trypanosome-host interactions revealed through transcriptomic analysis of parasitized tsetse fly salivary glands. PLoS Negl Trop Dis 8: e2649.
