# Supplementary material for: Uncovering Genomic Regions Associated with Trypanosoma Infections in Wild Populations of the Tsetse Fly Glossina fuscipes
Source: G3 (Bethesda). 2018 Jan 17;8(3):887–97. doi: 10.1534/g3.117.300493 (PMC5844309; doi:10.1534/g3.117.300493)
Supplement: Supplementary file 8 [file 887TableS4.pdf]

Table S4. Mapping the new *Glossina morsitans* assembly to previous assembly*

**Number of transcripts**	**Frequency**	**Description**
12053	14.04%	Complete match between two assemblies
38632	45.00%	Potentially novel isoform/fragment: at least one splice junction is shared with a transcript in the previous assembly
1240	1.44%	Generic exonic overlap with a transcript in the previous assembly
27978	32.60%	Unknown, intergenic transcript in the previous assembly
5937	6.91%	Exonic overlap with transcripts in the previous assembly on the opposite strand
9	0.01%	Others

*85849 transcripts in the new *Gmm* assembly were mapped to the previous *Gmm* assembly (https://www.vectorbase.org/, Yale strain GmorY1.4 geneset in GTF). Frequency was calculated as the number of transcripts in each category divided by total number of transcripts (85849) in the new assembly.
