# Supplementary material for: Uncovering Genomic Regions Associated with Trypanosoma Infections in Wild Populations of the Tsetse Fly Glossina fuscipes
Source: G3 (Bethesda). 2018 Jan 17;8(3):887–97. doi: 10.1534/g3.117.300493 (PMC5844309; doi:10.1534/g3.117.300493)
Supplement: Supplementary file 9 [file 887TableS5.docx]

**Table S5.** Differentially expressed genes identified by edgeR and Cuffdiff between infected and normal *Glossina morsitans* tissues.

| A. Number of differentially expressed (DE) genes identified from edgeR and Cuffdiff, respectively | | | | | |
| --- | --- | --- | --- | --- | --- |
| Tissue | **Parasites** | **edgeR** | | **Cuffdiff** | |
|  |  | **DE genes^2^** | **New DE genes^3^** | **DE genes** | **New DE genes** |
| Midgut | T. b. brucei | 3898 (2373, 1525) | 472(362, 110) | 4005 (1229, 2776) | 640 (332, 308) |
| Salivary Glands | T. b. brucei | 3876 (1900, 1976) | 1532 (563, 969) | 4848 (2883, 1965) | 2398 (1234,1164) |
| Proventriculus (1) | *T. congolense* | 128 (35, 93) | 8 (4, 4) | 151 (49, 102) | 27 (13, 14) |
| Proventriculus (2) | T. b. brucei | 855 (482, 373) | 179 (112, 67) | 682 (401, 281) | 195(117, 78) |
| Proventriculus (3)^1^ | T. b. brucei | 989 (761, 228) | 240 (183, 57) | 759 (411, 348) | 288 (163, 125) |
| Proboscis | T. b. brucei | 4848 (2923, 1925) | 3070 (2116, 954) | 4223 (2040,2183) | 2788 (1625, 1163) |

| B. Comparing edgeR with Cuffdiff results | | | | | | |
| --- | --- | --- | --- | --- | --- | --- |
| Tissue | **Upregulated in infected samples** | | | **Downregulated in infected samples** | | |
|  | **Both** | **edgeR** | **Cuffdiff** | **Both** | **edgeR** | **Cuffdiff** |
| Midgut | 930 | 1443 | 299 | 1184 | 341 | 1592 |
| Salivary Glands | 894 | 1006 | 1989 | 698 | 1278 | 1267 |
| Proventriculus (1) | 20 | 15 | 29 | 60 | 33 | 42 |
| Proventriculus (2) | 260 | 222 | 141 | 186 | 187 | 95 |
| Proventriculus (3)^1^ | 243 | 518 | 168 | 139 | 89 | 209 |
| Proboscis | 1054 | 1869 | 986 | 632 | 1293 | 1551 |

^1^. The three proventriculus tissues were collected from different experiments with different parasites and infectious status.

^2^. Number of differentially expressed genes (upregulated in infected samples, downregulated in infected samples)

^3^. Novel transcripts identified in the new *Gmm* assembly compared to the previous assembly (GmorY1.4).
