# Supplementary material for: Uncovering Genomic Regions Associated with Trypanosoma Infections in Wild Populations of the Tsetse Fly Glossina fuscipes
Source: G3 (Bethesda). 2018 Jan 17;8(3):887–97. doi: 10.1534/g3.117.300493 (PMC5844309; doi:10.1534/g3.117.300493)
Supplement: Supplementary file 11 [file 887TableS7.docx]

**Table S7:** Candidate loci associated with susceptibility to trypanosome infection in *Glossina fuscipes fuscipes* (*Gff*).

| ***Gff* loci^1^** | **Chrom^2^** | **SNP** | **Distance to gene (bp)** | **Name^3^** | **Dataset^4^** | **DE in Gmm^5^** |
| --- | --- | --- | --- | --- | --- | --- |
| Novel | Scaffold1 | 2366099 | -518 | XLOC_035548 | NB | T |
| Official | Scaffold101 | 665773 | 0 | GFUI002104 | NB | F |
| Official | Scaffold102 | 330092 | 0 | GFUI002233 | OT | F |
| Official | Scaffold102 | 398874 | 0 | GFUI002252 | OT | T |
| Official | Scaffold102 | 398874 | -1328 | GFUI002256 | OT | F |
| Official | Scaffold105 | 310781 | 1453 | GFUI002713 | MS_NB_OT | F |
| Official | Scaffold106 | 349187 | 0 | GFUI002898 | MS | F |
| Official | Scaffold107 | 284002 | 0 | GFUI003051 | OT | F |
| Official | Scaffold13 | 1603125 | 0 | GFUI007952 | OT | F |
| Official | Scaffold13 | 1931613 | -962 | GFUI008024 | MS | F |
| Official | Scaffold137 | 615407 | 0 | GFUI007441 | MS_NB_OT | T |
| Official | Scaffold144 | 593216 | 0 | GFUI008496 | MS_NB_OT | F |
| Novel | Scaffold151 | 406664 | 0 | *GMOY009422 | OT | T |
| Official | Scaffold151 | 406664 | -2155 | *GFUI009421 | OT | T |
| Official | Scaffold16 | 74447 | 0 | GFUI011180 | OT | F |
| Official | Scaffold16 | 74447 | 512 | GFUI011175 | OT | T |
| Official | Scaffold17 | 948302 | 0 | GFUI012390 | OT | F |
| Official | Scaffold175 | 347132 | 0 | GFUI011907 | MS_NB_OT | F |
| Official | Scaffold19 | 1333946 | 0 | GFUI014494 | NB | F |
| Novel | Scaffold2 | 1600828 | 98 | GMOY005234 | OT | T |
| Official | Scaffold2 | 1600828 | 0 | GFUI023675 | OT | F |
| Official | Scaffold2 | 2206337 | 0 | GFUI023751 | NB | T |
| Novel | Scaffold2 | 2370781 | 0 | GMOY005806 | MS_NB_OT | F |
| Novel | Scaffold22 | 1291805 | 0 | **GMOY004806 | MS | T |
| Official | Scaffold22 | 1291805 | 426 | **GFUI017734 | MS | T |
| Novel | Scaffold220 | 393433 | 0 | GMOY009338 | NB | T |
| Official | Scaffold24 | 1458470 | 1153 | GFUI019498 | OT | T |
| Novel | Scaffold268 | 383222 | 1700 | ^#^GMOY007568 | NB | T |
| Novel | Scaffold268 | 383222 | 1888 | GMOY007569 | NB | T |
| Official | Scaffold268 | 383222 | 0 | GFUI020790 | NB | T |
| Official | Scaffold268 | 383222 | 49 | GFUI020794 | NB | T |
| Official | Scaffold268 | 383222 | 1901 | ^#^GFUI020792 | NB | T |
| Official | Scaffold27 | 959995 | 0 | GFUI021833 | MS | T |
| Novel | Scaffold29 | 388431 | 0 | GMOY007432 | NB | F |
| Novel | Scaffold3 | 1688400 | 0 | GMOY010978 | MS_NB_OT | F |
| Official | Scaffold3 | 2235178 | 0 | GFUI030827 | MS | T |
| Novel | Scaffold33 | 120812 | -1406 | GMOY010556 | OT | F |
| Official | Scaffold33 | 120812 | 0 | GFUI026464 | OT | F |
| Official | Scaffold33 | 1387926 | 0 | GFUI026687 | MS_NB_OT | F |
| Official | Scaffold374 | 38707 | 733 | GFUI028910 | MS | T |
| Official | Scaffold40 | 697327 | 0 | GFUI031364 | OT | F |
| Official | Scaffold48 | 1013373 | 0 | GFUI035780 | MS_NB_OT | F |
| Official | Scaffold5 | 2342538 | 0 | GFUI041241 | OT | T |
| Novel | Scaffold64 | 729352 | 721 | ^##^GMOY005879 | NB | T |
| Official | Scaffold64 | 729352 | 0 | GFUI043027 | NB | T |
| Official | Scaffold64 | 729352 | 750 | ^##^GFUI043025 | NB | T |
| Official | Scaffold64 | 961545 | -2174 | GFUI043067 | NB | F |
| Official | Scaffold66 | 864866 | 0 | GFUI043720 | NB | T |
| Novel | Scaffold67 | 72303 | 0 | GMOY005501 | OT | T |
| Official | Scaffold67 | 72303 | 1614 | GFUI043916 | OT | F |
| Official | Scaffold768 | 20355 | 1035 | GFUI047097 | MS | F |
| Official | Scaffold8 | 1744226 | 0 | GFUI051070 | MS_NB_OT | T |
| Novel | Scaffold9 | 1360708 | -2432 | ^&^GMOY003473 | NB | T |
| Official | Scaffold9 | 1360708 | 0 | ^&^GFUI053401 | NB | T |
| Official | Scaffold9 | 2033837 | 0 | GFUI053502 | OT | F |
| Official | Scaffold94 | 691028 | 1826 | GFUI052240 | OT | F |

^1^ Novel: novel mapped Tx; Official: official annotation.

^2^ Genomic scaffold in which the SNP is located (GfusI1.3).

^3^ Name of closest gene to the SNP. GFUI loci are those genes annotated in the GfusI1.3 genome; GMOY loci represent the homologous annotated gene in *Glossina morsitans* (GmorY1.4) for that novel *Gff* gene; XLOC indicates that the gene is not annotated in neither the *Gff* or *Gmm* official assemblies.

^4^ SNP dataset on which the genetic association test was performed.

^5^ Expression status in *Glossina morsitans* between infected and uninfected flies. T: TRUE, the transcript is differentially expressed; F: False, the transcript is NOT differentially expressed.

Symbols before the gene name indicate possible paralog genes (*, **, #,##, and & ).
