# Supplementary material for: Uncovering Genomic Regions Associated with Trypanosoma Infections in Wild Populations of the Tsetse Fly Glossina fuscipes
Source: G3 (Bethesda). 2018 Jan 17;8(3):887–97. doi: 10.1534/g3.117.300493 (PMC5844309; doi:10.1534/g3.117.300493)
Supplement: Supplementary file 12 [file 887TableS8.docx]

**Table S8.** Comparison of differentially expressed genes across tissues

| **Methods** | **Differential expression** | **Total unique genes** | **Shared across all tissues** | **Shared by >=2 tissues** |
| --- | --- | --- | --- | --- |
| cuffdiff | Up | 6306 | 0 | 609 |
|  | Down | 6887 | 0 | 653 |
| edgeR | Up | 7244 | 0 | 1000 |
|  | Down | 5390 | 0 | 616 |
| Both | Up | 3064 | 0 | 278 |
|  | Down | 2650 | 0 | 243 |
